# Risk of subsequent gastrointestinal disease assessed by skeletal muscle strength and mass in a prospective cohort study

**DOI:** 10.1016/j.isci.2024.109341

**Published:** 2024-03-05

**Authors:** Lintao Dan, Pei Qin, Siyuan Xie, Yuhao Sun, Tian Fu, Xixian Ruan, Wenming Shi, Jie Chen, Jianting Cai, Xue Li

**Affiliations:** 1 Department of Big Data in Health Science, School of Public Health and The Second Affiliated Hospital, Zhejiang University School of Medicine, Hangzhou, China; 2 Shenzhen Qianhai Shekou Free Trade Zone Hospital, Shenzhen, Guangdong, China; 3 Department of Gastroenterology, The Second Affiliated Hospital, Zhejiang University School of Medicine, Hangzhou, China; 4 Department of Gastroenterology, The Third Xiangya Hospital, Central South University, Changsha, China; 5 School of Public Health, Li Ka Shing Faculty of Medicine, The University of Hong Kong, Hong Kong SAR, China

**Keywords:** Gastroenterology, Public health, Risk stratification

## Abstract

Skeletal muscle may mutually interact with gastrointestinal disease through metabolic homeostasis and nutritional status and therefore may be a marker for early risk detection. We conducted a prospective cohort analysis including 393,606 participants (mean age 56.0 years, 53.9% female) from the UK Biobank. The exposures were grip strength and skeletal muscle mass (SMM). The primary outcomes were 24 incident gastrointestinal diseases. During a mean follow-up of 12.1 years, we found that one sex-specific SD increase in grip strength and SMM were associated with reduced risk of 16 and 19 gastrointestinal diseases, respectively. For grip strength, the HRs ranged from 0.94 (for ulcerative colitis) to 0.80 (for liver cancers). For SMM, the HRs ranged from 0.92 (for colorectal cancer) to 0.51 (for non-alcoholic fatty liver disease). Our finding suggested that grip strength and SMM might be significant indicators for gastrointestinal diseases risk screen.

## Highlights

• Higher grip strength was associated with reduced risk of 16 gastrointestinal diseases
• Higher muscle mass was associated with reduced risk of 19 gastrointestinal diseases
• Reveal unreported associations of muscle indicators with 10 gastrointestinal diseases
• Highlight the potential of muscle indicators as early risk screen manners

## Graphical abstract

**Figure:**
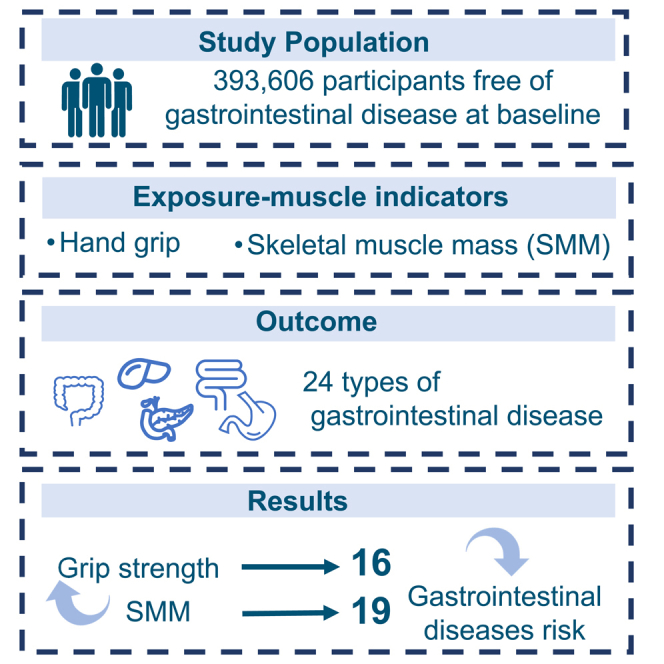

## Introduction

Gastrointestinal diseases affect people of all ages and lead to substantial healthcare utilization. It is estimated that approximately 2.3 billion people suffering from gastrointestinal diseases and 2.6 million deaths were attributed to gastrointestinal diseases in 2019 according to Global Burden of Disease study.^1^ To identify high-risk groups and develop early screening strategies for gastrointestinal diseases, it is crucial to identify new risk factors.

Grip strength and skeletal muscle mass (SMM) are solid indicators of the quality and quantity of skeletal muscle. They have been reported as early indicators of multiple chronic diseases including type 2 diabetes, cardiovascular diseases, and cancers.^2^^,^^3^^,^^4^ Skeletal muscle indicators may also be used for early identification of gastrointestinal diseases. On the one hand, skeletal muscle involves the maintenance of glucose homeostasis and metabolic health^5^ and has the potential to cause gastrointestinal problems. Recent studies have systematically revealed the association of diabetes, obesity, and other factors related to body composition and metabolic homeostasis with gastrointestinal diseases.^6^^,^^7^ On the other hand, gastrointestinal diseases can affect skeletal muscle via nutrition status,^8^ and changes in skeletal muscle may precede the clinical diagnosis of gastrointestinal diseases and may be an indicator of early detection.

However, whether skeletal muscle status can serve as an early indicator for subsequent gastrointestinal diseases remains unsatisfactorily answered. Current studies from several large cohorts have added to our understanding of the association of grip strength and muscle mass with gastrointestinal cancers and liver disease,^2^^,^^4^^,^^9^^,^^10^ but there are inconsistencies in these results. Also, there is limited data on their association with other gastrointestinal diseases. Therefore, more evidence from large prospective cohorts that take into account chronological order is important to inform decisions on public health interventions.

In this study, we aimed to systematically assess the associations of grip strength and SMM with a spectrum of gastrointestinal diseases in the UK Biobank cohort study.

## Results

### Baseline characteristics

Table 1 showed the main characteristics of the individuals by tertiles of grip strength and SMM, respectively. The mean age at recruitment was 56.0 (standardized deviation [SD] 8.1) years and 53.9% were female. Individuals with the highest tertiles of grip strength and SMM were likely to have higher levels of physical activity, never smoked, and had lower alcohol consumption.

**Table 1:** Baseline characteristics of study sample at baseline visit according to grip strength and appendicular skeletal muscle mass in tertiles^a^

	Overall	Grip strength			Appendicular skeletal muscle mass		
	(n = 393,606)	Lowest	Middle	Highest	Lowest	Middle	Highest
		(n = 139,100)	(n = 124,611)	(n = 129,845)	(n = 131,213)	(n = 131,203)	(n = 131,190)
Grip (mean (SD)), kg	31.05 (11.0)	23.2 (7.9)	31.4 (8.2)	39.1 (10.3)	–	–	–
Appendicular muscle mass/weight (mean (SD))	0.29 (0.04)	–	–	–	0.26 (0.03)	0.29 (0.03)	0.31 (0.03)
Age at baseline (mean (SD))	56.0 (8.1)	58.6 (7.6)	56.4 (7.9)	53 (7.9)	58.6 (7.5)	56.5 (7.8)	53.1 (8.1)
Sex (%)							
Female	211,984 (53.9)	76,013 (54.6)	66,382 (53.3)	69,589 (53.6)	70,518 (53.7)	69,733 (53.1)	71,733 (54.7)
Male	181,622 (46.1)	63,087 (45.4)	58,279 (46.7)	60,256 (46.4)	60,695 (46.3)	61,470 (46.9)	59,457 (45.3)
Townsend deprivation index (mean (SD))	−1.4 (3.1)	−1.1 (3.2)	−1.5 (3.0)	−1.6 (3.0)	−1.2 (3.2)	−1.5 (3.0)	−1.4 (3.0)
Education (%)							
Below college degree	254,863 (65.5)	96,026 (70.2)	80,422 (65.2)	78,415 (60.8)	95,637 (73.9)	85,816 (66.1)	73,410 (56.5)
College degree	134,312 (34.5)	40,848 (29.8)	42,981 (34.8)	50,483 (39.2)	33,754 (26.1)	43,954 (33.9)	56,604 (43.5)
Ethnicity (%)							
White	369,834 (94.4)	128,180 (92.7)	118,311 (95.3)	123,343 (95.3)	122,826 (94.1)	123,534 (94.6)	123,474 (94.5)
Others	21,916 (5.6)	10,068 (7.3)	5826 (4.7)	6022 (4.7)	7730 (5.9)	7064 (5.4)	7122 (5.5)
Physical activity (%)							
Inadequate	115,401 (29.3)	47,519 (34.2)	35,623 (28.6)	32,259 (24.8)	49,583 (37.8)	37,083 (28.3)	28,735 (21.9)
Adequate	278,205 (70.7)	91,581 (65.8)	89,038 (71.4)	97,586 (75.2)	81,630 (62.2)	94,120 (71.7)	102,455 (78.1)
Smoking status (%)							
Never smoked	219,658 (56.1)	77,193 (55.9)	69,160 (55.7)	73,305 (56.6)	66,526 (51.0)	73,791 (56.5)	79,341 (60.7)
Previous or current smokers	172,093 (43.9)	60,979 (44.1)	54,993 (44.3)	56,121 (43.4)	63,905 (49.0)	56,792 (43.5)	51,396 (39.3)
Charlson comorbidity index (mean (SD))	0.2 (0.8)	0.3 (0.9)	0.2 (0.8)	0.1 (0.6)	0.3 (0.9)	0.2 (0.7)	0.2 (0.7)
Alcohol consumption (%)							
None to moderate consumption	311,476 (79.3)	113,409 (81.8)	97,995 (78.7)	100,072 (77.2)	102,785 (78.5)	102,933 (78.6)	105,758 (80.8)
Heavy consumption	81,337 (20.7)	25,230 (18.2)	26,487 (21.3)	29,620 (22.8)	28,120 (21.5)	28,035 (21.4)	25,182 (19.2)
BMI (mean (SD))	27.2 (4.7)	27.4 (4.9)	27.1 (4.6)	27.3 (4.6)	31.2 (4.7)	26.8 (2.9)	23.7 (2.8)
Healthy diet (%)							
Unhealthy	105,392 (28.1)	37,172 (28.6)	32,892 (27.6)	35,328 (28.2)	38,897 (31.5)	34,654 (27.7)	31,841 (25.3)
Healthy	269,050 (71.9)	92,915 (71.4)	86,231 (72.4)	89,904 (71.8)	84,493 (68.5)	90,480 (72.3)	94,077 (74.7)

BMI, body mass index.

a Mean (SD) values and percentages are reported for continuous and categorical variables, respectively.

### Associations between grip strength and gastrointestinal diseases

**Figure 1:**
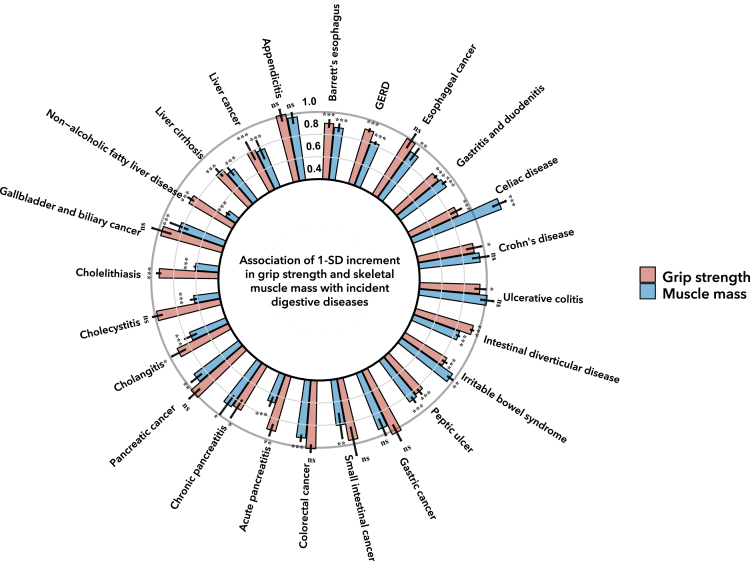
The associations of grip strength and skeletal muscle mass (1-SD increment) and risk of twenty-four gastrointestinal diseases The error bars represent the 95% CIs for corresponding HRs. The HR and 95% CI was estimated based on the fully adjusted model. Q value was the corrected p value after multiple comparisons. ∗, Q < 0.05; ∗∗, Q < 0.01; ∗∗∗, Q < 0.001; ns, not significant. CI, confidence interval; GERD, Gastroesophageal reflux disease; HR, hazard ratio.

During a mean follow-up of 12.1 years, after multivariable adjustment and correction for multiple comparisons, we found higher grip strength (per 1-SD increment) was significantly associated with decreased risk of 16 gastrointestinal diseases, including Barrett’s esophagus, gastroesophageal reflux disease (GERD), gastritis and duodenitis, celiac disease, Crohn’s disease, ulcerative colitis, intestinal diverticular disease, irritable bowel syndrome, peptic ulcer, acute pancreatitis, chronic pancreatitis, cholangitis, cholelithiasis, non-alcoholic fatty liver disease (NAFLD), liver cirrhosis, and liver cancer (Figure 1). The Hazard ratios (HRs) ranged from 0.80 (95% confidence interval [CI] 0.72–0.89, false discovery rate corrected p value [Q-value] <0.001) for liver cancer to 0.94 (95% CI 0.90–0.99, Q = 0.044) for ulcerative colitis. The detailed results were presented in Table S1. No significant associations were found for esophageal cancer, gastric cancer, small intestinal cancer, colorectal cancer, pancreatic cancer, cholecystitis, gallbladder and biliary cancer, and appendicitis (All Q > 0.05).

**Figure 2:**
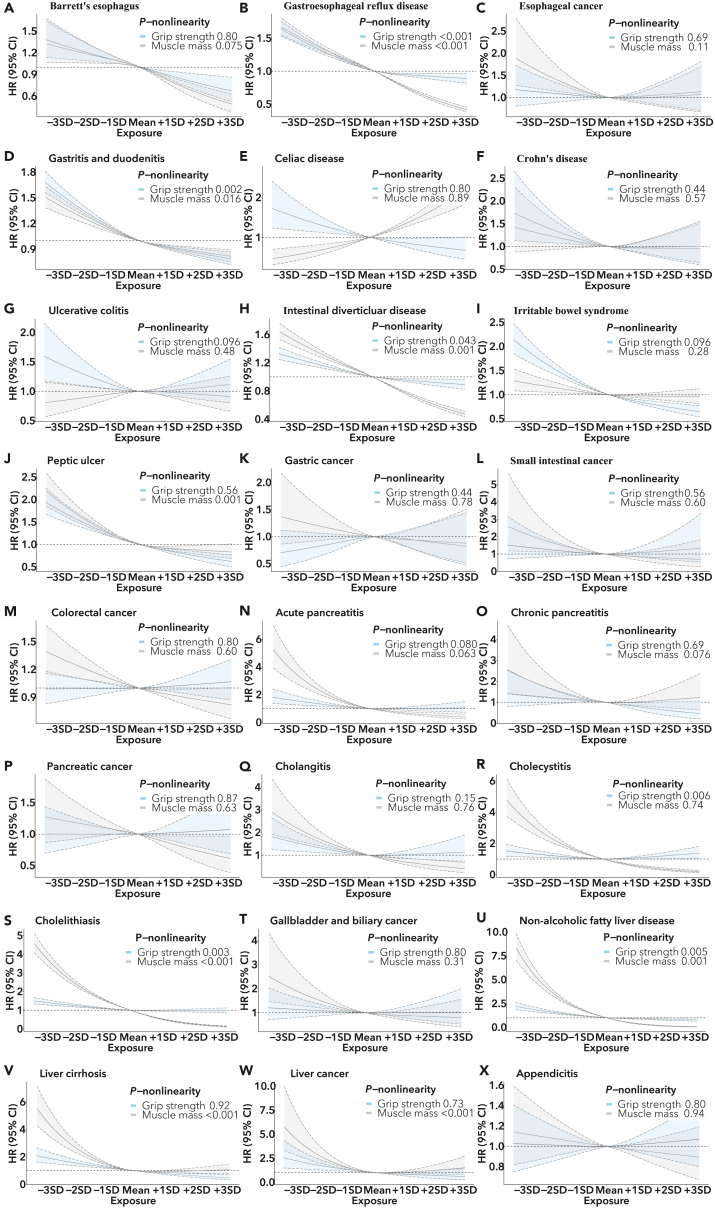
RCS curves for the associations of grip strength and skeletal muscle mass and risk of twenty-four gastrointestinal diseases (A) Barrett’s esophagus, (B) gastroesophageal reflux disease, (C) esophageal cancer, (D) gastritis and duodenitis (F) celiac disease, (F) Crohn’s disease, (G) ulcerative colitis, (H) intestinal diverticular disease, (I) irritable bowel syndrome, (J) peptic ulcer, (K) gastric cancer, (L) small intestinal cancer, (M) colorectal cancer, (N) acute pancreatitis, (O) chronic pancreatitis, (P) pancreatic cancer, (Q) cholangitis, (R) cholecystitis, (S) cholelithiasis, (T) gallbladder, and biliary cancer (U) non-alcoholic fatty liver disease, (V) chronic liver disease/cirrhosis, (W) liver cancers (X) and appendix diseases. Curves in blue and gray represent grip strength and skeletal muscle mass, respectively. *P*-nonlinearity had been corrected with FDR control. The HR and 95% CI was estimated based on the fully adjusted model. CI, confidence interval; HR, hazard ratio.

Dose-response associations the change curve in the risk of gastrointestinal diseases corresponding to grip strength among individuals in the study sample were presented in Figure 2. We observed inverse associations consistent with the results of the categorical model, while nonlinearity was detected for GERD, gastritis and duodenitis, intestinal diverticular disease, cholecystitis, cholelithiasis, and NAFLD (All p value for nonlinearity <0.05).

### Associations between SMM and gastrointestinal diseases

As shown in Figure 1 and Table S1, after multivariable adjustment and correction for multiple comparisons, we found 1-SD increase in SMM was associated with a 25% (95% CI 19%–31%) increased risk of celiac disease and reduced risk of 19 gastrointestinal diseases including Barrett’s esophagus, GERD, esophageal cancer, gastritis and duodenitis, celiac disease, intestinal diverticular disease, irritable bowel syndrome, peptic ulcer, small intestinal cancer, colorectal cancer, acute pancreatitis, chronic pancreatitis, pancreatic cancer, cholangitis, cholecystitis, cholelithiasis, gallbladder and biliary cancer, NAFLD, liver cirrhosis, and liver cancer (Figure 1). The HR of grip strength ranged from 0.51 (95% CI 0.49–0.52, Q < 0.001) for non-alcoholic fatty liver to 0.92 (95% CI 0.90–0.95, Q < 0.001) for colorectal cancer. No significant associations of SMM with Crohn’s disease, ulcerative colitis, gastric cancer, and appendicitis were observed (All Q > 0.05).

As shown in Figure 2, we observed dose-response associations consistent with the results of the categorical model, while nonlinearity was detected for GERD, gastritis and duodenitis, intestinal diverticular disease, peptic ulcer, cholelithiasis, NAFLD, liver cirrhosis, and liver cancer (All p value for nonlinearity <0.05). Consistent positive dose-response associations between SMM and celiac disease were observed.

### Secondary and subgroup analysis

As shown in Tables S2 and S3, assuming causality, the proportion of disease incidence attributable to having the lowest tertiles of grip and SMM ranged from 3.4% to 15.9% and 4.0%–38.2%, respectively. In our study, the corresponding gender-specific tertile cutoff values for SMM were 0.31 kg (males) and 0.25 kg (females) per 1 kg of body weight, and the gender-specific tertile cutoff values for grip strength thresholds were 36 kg (males) and 21 kg (females). Most of the associations of SMM with gastrointestinal diseases were consistent when using SMM/weight, SMM/body mass index (BMI), or SMM/fat mass as exposure to adjust the impact of body size on SMM (Table S4). In subgroup analysis, a significant interaction between age and grip strength was observed for esophageal cancer, gastritis and duodenitis, and liver cirrhosis (Table S5, all p value for interaction <0.05), and the associations were broadly similar for other outcomes. There was a significant interaction between sex and grip strength for GERD, intestinal diverticular disease, irritable bowel syndrome, and NAFLD (Table S5, all p value for interaction <0.05). The associations were similar for the subgroup analysis by alcohol consumption, smoking status, and diet (Table S6).

Subgroup analysis also showed that age, gender, alcohol consumption, smoking status, and diet significantly interacted with SMM in relation to 10, 12, 2, 7, and 3 gastrointestinal diseases, respectively (Tables S7 and S8, all p value for interaction <0.05). Among them, we found the most significant difference in the estimated protective association between increased SMM and GERD risk among males and females (HR 0.91 vs. 0.76, p value for interaction <0.001).

### Sensitivity analysis

The primary findings remained robust in sensitivity analyses by excluding incident cases in the first 3-year follow-up (Table S9), excluding individuals with extreme values (1st or 99th quantile) of exposures (Table S10), further adjusting for inflammatory indicators (Table S11) or excluding individuals with missing values (Table S12). The association of grip strength (by 1-SD increment) with Crohn’s disease (HR 0.93, 95% CI 0.86–1.01, Q = 0.16) and ulcerative colitis (HR 0.95, 95% CI 0.90–1.01, Q = 0.16) became non-significant after exclusion of incident cases in the first 3-year follow-up. Certain associations were amplified slightly in the analysis among the propensity score-matched population (Table S13).

## Discussion

In this prospective study of 393,606 UK adults, we systematically assessed the associations of grip strength and SMM with the subsequent risk of 24 gastrointestinal diseases. Our study provided supportive evidence on the associations of grip strength and SMM with GERD, liver disease, and gastrointestinal cancers as reported in previous studies. We also revealed associations of grip strength and SMM with other 10 gastrointestinal diseases, which have not been reported or examined. The robustness of these findings was further tested by a series of sensitivity analyses.

The associations of grip strength and SMM with GERD and liver disease were in line with previous studies. One cross-sectional study covering 542 old people found that 5 kg increase in grip strength was associated 17% lower risk of GERD in 542 older adults,^11^ while sarcopenia, a skeletal muscle attenuation status, was found to be positively associated with an increased risk of in two small Asian health check-up cohorts.^12^^,^^13^ For liver disease, a prospective cohort study conducted in a general Chinese population (n = 14,154) found that grip strength was inversely associated with the incidence of NAFLD (HR for comparing extreme quartiles of grip: 0.44 for men; 0.41 for women).^14^ A recent study using the UK biobank data found similar results of the significant association between SMM and grip strength with severe NAFLD.^15^ Previous studies also found grip strength was found to be a predictor of mortality in patients with cirrhosis.^16^

For gastrointestinal cancer, in our studies, higher grip strength was only significantly associated with reduced risk of liver cancer while was not associated with esophageal, stomach, small intestine, colorectal, pancreas, and biliary cancer. A previous study using the UK biobank data also found non-significant associations between grip strength and stomach, colorectal, or pancreatic cancer and a significant inverse association for liver cancer.^4^ We have extended limited evidence in the association between SMM and gastrointestinal cancers, finding a decreased risk of esophageal, small intestinal, colorectal, and pancreatic cancer for higher SMM, but not risk of gastric cancer. The findings were relatively consistent with previous studies showing that low SMM is associated with high morbidity and mortality from esophageal,^17^ and colorectal cancer.^18^ A recent review also showed that low SMM is endemic across a range of gastrointestinal cancers.^19^ These results suggested that SMM might be a more appropriate indicator than grip strength in terms of identifying populations at high risk for gastrointestinal cancer.

Our study also provided novel evidence for the development of Barrett’s esophagus and non-cancer disease of the stomach, bowel, pancreas, gallbladder, biliary tract, and appendix. To our knowledge, there is no observational evidence that has linked grip strength and SMM to the development of these diseases. However, several studies provided indirect evidence to plausibly support the associations reported in the present study. One prospective study found that frailty, defined by five criteria including low grip strength, was associated 80% increased risk of irritable bowel syndrome.^20^ Another study using data from the Korea National Health and Nutrition Examination Survey revealed a positive association between sarcopenia and peptic ulcer.^21^ In addition, decreased grip strength and SMM, as the key components of frailty or sarcopenia, were reported to be more prevalent and associated with worse prognosis in individuals with Crohn’s disease, ulcerative colitis, celiac disease, pancreatitis, and gallbladder and biliary disease.^22^^,^^23^^,^^24^^,^^25^

Although studies examining the association of grip strength and SMM with gastrointestinal disease are limited, the current accumulating evidence implies a mechanistic rationale for predicting the onset of gastrointestinal disease by skeletal muscle indicators. In the context of the skeletal muscle-gut axis,^26^ attenuation in both the quality and quantity of skeletal muscle are related to increased levels of circulating oxidative stress markers and dysbiosis of aging-related gut microbiota.^27^^,^^28^ This alteration will lead to increased intestinal permeability, facilitating the passage of endotoxin and other microbial products.^26^ Moreover, skeletal muscle fiber enables the uptake of glucose via glucose transporter type 4 and muscle attenuation can induce insulin intolerance, which is a component of metabolic syndrome. Two Mendelian randomization studies have systematically revealed casual associations of diabetes and obesity with more than 20 gastrointestinal diseases,^6^ which implies that skeletal muscle may play a causal role in the development of gastrointestinal diseases via disturbance in metabolic homeostasis.

We also observed, in the subgroup analysis, the associations of grip and SMM with gastrointestinal diseases were generally stronger in older people than in younger ones, and males than in females. This age and sex difference may be also due to differences in body composition, upper body strength, lifestyle patterns, sex hormones, and inflammatory load.^29^^,^^30^ Men have more rapid age declines in muscle strength, mass, and quality compared with women,^31^ which may be one reason for the sex differences. Future studies are needed to elucidate reasons for these age and gender differences as well as the interaction with lifestyle factors.

For those associations that were significant in the categorical model, visualized dose-response confirmed that the corresponding risk of gastrointestinal diseases progressively declined or reached a plateau as grip strength and muscle increased, regardless of the presence of nonlinearity. The only exception is celiac disease, while inversely associated with grip strength, it is positively associated with the skeletal muscle mass represented by SMM/weight, SMM/BMI, or SMM/fat mass. One possible explanation is malnutrition or malabsorption associated with celiac disease. A questionnaire survey conducted in Poland, which included 572 adult patients, showed that 58% and 43.6% of patients experienced diarrhea and weight loss before the diagnosis of celiac disease, respectively, with an average duration of 5.8 and 6.4 years^32^ This means that patients may be suffering from malnutrition and related loss of muscle strength and quantity long before diagnosis, making it reasonable to use muscle indicators for early screening for gastrointestinal disorders. However, as a disease characterized by lesions of the small intestinal mucosa that directly affect nutrient absorption, it is unknown whether the weight loss caused by celiac disease is specifically a loss of adipose tissue or muscle tissue.^33^ Steatorrhea is a key feature of older-onset celiac disease patients, which may imply that the weight loss in the long period before diagnosis is primarily a reduction in adipose tissue (i.e., the proportion of muscle to total body weight is instead elevated in a state of weight loss). Overall, no previous studies have explored differences in body composition before the diagnosis in celiac disease patients compared to the general population, and we are currently in the speculative stage of interpreting the results demonstrating a positive association between SMM and the onset of celiac disease. Further studies like the Mendelian randomization study are needed to clarify the association between muscle mass and the onset of celiac disease.

Our study revealed an elevated risk of multiple gastrointestinal diseases in the individuals with the lowest tertile of grip strength and SMM(important indicators for the diagnosis of sarcopenia), compared to those with higher levels. Our findings suggest that individuals with decreased muscle mass/loss of grip strength/suspected sarcopenia found in clinical and routine physical exams/screening compared to area-specific reference values need to be concerned about digestive-related symptoms, biomarkers, and examinations to rule out the risk of digestive disorders. Future research should address the optimal categorization of muscle mass and grip strength so that grip strength and muscle mass can be used as routine risk indicators for gastrointestinal disease in public health practice.

Calculation of attributable fraction (AF) showed that if the grip strength or muscle mass levels of individuals currently in the lowest tertile of the population could be increased by means of interventions, then 3.4%–38.2% of the corresponding gastrointestinal diseases could potentially be avoided under the assumption that causality is established. Although based on relatively ideal assumptions, this ratio provides a preliminary estimate of the possible benefits of improving the muscle indicators for gastrointestinal disease prevention. Therefore, our research encourages interventional studies to investigate whether the prevention of gastrointestinal disorders can be achieved by intervening in grip strength and muscle mass.

The strength of our study lies in the prospective design, which clarified the chronological order of events. Secondly, we performed several statistical analysis strategies, not only to give results for the magnitude of the associations but also to draw dose-response curves to make a quantitative description. Finally, the study outcomes covered almost all common gastrointestinal diseases, providing a more comprehensive exploration of the research question.

Overall, we found that increased quantity and quality of muscle mass were associated with subsequent reduced risk of a broad spectrum of gastrointestinal diseases. Our study with a longitudinal design highlighted the potential of low levels of grip strength and muscle mass within the population as early screening indicators for the development of gastrointestinal disease. More future studies are needed to confirm our results and the effectiveness of skeletal muscle examination as a means of early screening for gastrointestinal disease in a longitudinal frame. Also, studies clarifying whether improvement of muscle mass and strength reduces gastrointestinal diseases are important for public health.

### Limitations of the study

Firstly, the study population was predominantly non-Hispanic white, so considerations should be taken in generalizing the results to the general population. Secondly, reverse causality is possible in any observational study but our design excluded individuals with any of 24 gastrointestinal diseases at baseline or within the first 1-year follow-up and results were similar after the sensitivity analysis of events occurring in the first 3-year follow-up. Thirdly, although we did not use dual-energy X-ray absorptiometry as the most accurate method for measuring muscle mass, bioelectrical impedance analysis was widely used in large population studies as it was an inexpensive and easily portable approach.^34^ Muscle mass estimated using bioelectrical impedance analysis has been shown to have a high agreement with dual-energy X-ray absorptiometry in the UK Biobank.^35^ Moreover, unmeasured or residual confounding cannot be avoided despite adjusting for many potential confounding factors, and we applied the propensity score matching method to further address this concern.

## STAR★Methods

### Key resources table

**Table:** 

REAGENT or RESOURCE	SOURCE	IDENTIFIER
**Deposited data**		

UK Biobank	https://www.ukbiobank.ac.uk	Application number 66354

**Software and algorithms**		

R	https://www.r-project.org	Version 4.1.1
Original code	https://zenodo.org/records/10663543	https://doi.org/10.5281/zenodo.10663543

### Resource availability

#### Lead contact

Further information and requests for resources should be directed to and will be fulfilled by the Lead Contact, Xue Li (xue.li@ed.ac.uk).

#### Materials availability

This study did not generate new unique reagents.

#### Data and code availability

• This paper analyzes existing, publicly available data. These accession numbers for the datasets are reported in the content above.
• All original code has been deposited at https://zenodo.org/records/10663543 and is publicly available as of the date of publication (https://doi.org/10.5281/zenodo.10663543).
• Any additional information required to reanalyze the data reported in this paper is available from the lead contact upon request.

### Experimental model and study participant details

#### Study population

This study leveraged data from the UK Biobank, which was an ongoing national prospective cohort project that enrolled over 500,000 volunteers from 22 assessment centers across the UK during 2006-2010. At baseline, each participant received a touch-screen questionnaire, a brief computer-assisted interview, physical measurements, and laboratory tests. All individuals have signed an electronic consent, and the North West–Haydock Research Ethics Committee granted ethical approval to use the UK Biobank database (REC reference: 21/NW/0157). In this study, we excluded (1) individuals with unavailable information about grip strength or SMM (*n* = 10,875); (2) individuals with any of 24 gastrointestinal diseases at baseline or within the first 1-y follow-up to reduce potential reverse causality (*n* = 97,980). After exclusions, 393,606 individuals were included in our analysis (Figure S1). The mean age at recruitment was 56.0 (SD 8.1) years and 53.9% were female. The biological sex of participants was confirmed by the records from National Health Service and self-reported information. The self-reported ethnic background of the study sample included White, British, Irish, any other white background, mixed, Asian or Asian British, Black or Black British, Chinese, and other ethnic groups (not listed above). This study followed the Strengthening the Reporting of Observational Studies in Epidemiology guideline.

### Method details

#### Measurement of grip strength and SMM

As recommended by the European Working Group on Sarcopenia in Older People (EWGSOP2), grip strength and SMM were adopted as validated indicators for muscle strength and quantity.^36^ Grip strength was measured using a Jamar J00105 hydraulic hand dynamometer. Mean values of grip strength of both hands were calculated in subsequent analyses. SMM was measured by bioelectrical impedance analysis based on the Tanita BC418MA Body Fat Analyzer (Tanita Corporation, Arlington Heights, IL). SMM was the sum of the predicted skeletal muscle mass of the arms and legs. Appendicular SMM accounts for approximately 75% of whole-body SMM in adults and is the most modifiable fraction of whole-body skeletal muscle mass.^37^ The measurements obtained from bioelectrical impedance analysis and dual-energy X-ray absorptiometry for SMM demonstrated high correlations (intraclass correlation coefficient > 0.8) in a subset of the UK Biobank.^35^ Both exposures were standardized using sex-specific mean and standard deviation of the whole sample as recommended by the previous study^4^ and then categorized by tertiles. For grip strength, a study in the UK Biobank previously demonstrated that the association of grip strength with health outcomes did not differ whether it was expressed in absolute or relative terms.^38^ For the SMM, we divided it by weight (SMM/weight) to adjust for body size, as suggested by EWGSOP2.^36^

#### Ascertainment of outcomes

The outcomes of interest were twenty-four gastrointestinal diseases, including esophagus diseases (Barrett’s esophagus, GERD, esophageal cancer), stomach and bowel diseases (gastritis and duodenitis, celiac disease, Crohn’s disease, ulcerative colitis, intestinal diverticular disease, irritable bowel syndrome, peptic ulcer, gastric cancer, small intestinal cancer, colorectal cancer), pancreas diseases (acute pancreatitis, chronic pancreatitis, pancreatic cancer), gallbladder and biliary diseases (cholangitis, cholecystitis, cholelithiasis, gallbladder, and biliary cancer), liver diseases (NAFLD, chronic liver disease/cirrhosis, liver cancers [hepatocellular carcinoma and intrahepatic cholangiocarcinoma]), and appendix diseases (appendicitis). The UK Audit Commission^39^ review of 2009 to 2010 concluded single diagnostic coding ICD-10 overall accuracy of 89%.

Diseases were considered individually and incident diagnoses were defined as the first record of that condition in inpatient data, primary care data, and cancer registry. Diagnostic codes for each disease are presented in Tables S14 and S15. For each outcome, follow-up time was calculated from the time of recruitment to the time of the diagnosis of the corresponding disease, death, or end of follow-up (September 2021 for individuals in England, July 2021 for individuals in Scottish, and February 2018 for individuals in Wales), whichever occurred first.

#### Assessment of covariates

Covariates were predefined and details for assessment and missing rate were presented in Table S16. Information including age, sex (female, male), ethnic background (white, non-white), education quantification, smoking status (never, previous or current), alcohol consumption (none to moderate, heavy), and physical activity (adequate and inadequate) was obtained from touch screen questionnaires. Ethnicity was categorized into “White” (White, British, Irish, and any other white background) and “Others” (Mixed, Asian or Asian British, Black or Black British, Chinese, and other ethnic groups) based on the self-reported items. The Townsend deprivation index was used as a measurement of socioeconomic deprivation, which was automatically calculated from the postcodes of the UK Biobank individuals. The BMI was calculated using height and weight in the physical measurement. Physical activity was collected using a validated short International Physical Activity Questionnaire and assessed as adequate or inadequate based on the recommendation from the American Heart Association.^40^ Adequate physical activity was defined as 150 min moderate activity per week, or ≥ 75 min vigorous activity per week, or equivalent combination, or moderate physical activity at least 5 days a week or vigorous activity once a week. Alcohol consumption was estimated via 19-item touchscreen questionnaires that were described before.^41^ None to moderate level of alcohol consumption was defined as 0-14 g/d for women and 0-28 g/d for men according to US dietary guidelines,^42^ above which is defined as heavy level. Charlson Comorbidity Index was included as a measure of comorbidity and objective health status.^43^ Diet quality was assessed by a 7-item healthy diet score with data from food frequency questionnaires.^44^ Two indicators, C-reactive protein, and INFLA-score were selected to represent systematic inflammation, The INFLA-score contained C-reactive protein, white blood cell, platelet count, and the neutrophil-to-lymphocyte ratio, synergistically having a pro-inflammatory role in different biological processes of the immune response.^45^ If covariate information was missing or recorded as “unknown”, we imputed the median values for continuous variables or applied a most frequently used category for categorical variables.

### Quantification and statistical analysis

#### Statistical analysis

Baseline characteristics by tertiles of grip strength and SMM were presented as means (SDs) for continuous variables and numbers (percentages) for categorical variables. Cox proportional hazards regression using age as the time scale was applied to estimate HRs and 95% CIs for the association of grip strength and SMM with the risk of twenty-four diseases by 1-SD increment or tertiles. The proportional hazard assumptions of all Cox models were confirmed by visually assessing the scaled Schoenfeld residuals. Two multivariable models were constructed with confounders. The minimally adjusted model was adjusted for age, sex, and ethnicity. The fully adjusted model was further adjusted for Townsend deprivation index, smoking status, education, alcohol consumption, physical activity, BMI, healthy diet, and Charlson comorbidity index. To describe the dose-response associations and test the potential nonlinearity of the associations, restricted cubic splines (RCS) were used with three knots at the 10th, 50th, and 90th percentiles. The non-linearity of the spline curve was tested using the likelihood ratio test.

In the secondary analysis, we conducted analyses using SMM divided by whole body fat mass or BMI as exposure to further account for the impact of varied body size among individuals.^36^ To quantify the potential impact of grip strength and SMM on the incidence of gastrointestinal diseases, we estimated AF for those that demonstrated significant inverse associations between exposures and incident disease. AF was a semiparametric estimation for cohort studies when the outcome was time-to-event, indicating the proportion of disease incidence can be avoided under the hypothetical scenario where the lowest tertiles of grip strength or SMM levels were eliminated from the population (i.e., Raise those currently at the lowest tertile level above the corresponding tertile cutoff). The formula is

$$AF=1-\frac{\{1-S_{0}\left(t\right)\}}{\left\{1-S\left(t\right)\right\}}$$

where $S_{0}\left(t\right)$ denotes the counterfactual survival function for the event if the exposure would have been eliminated from the population at baseline and S(t) denotes the factual survival function. We estimated the AF using the “AFcoxph” function using the R package “AF”, which was described in detail elsewhere.^46^^,^^47^

We performed subgroup analyses for age, sex, alcohol consumption, smoking status, and healthy diet. The multiplicative interaction was calculated to find subgroups that may statistically benefit (if causal) more from exposure factors, evaluating by testing the change of models before and after allowing a multiplication term of the exposure and the covariates. For sensitivity analysis, we performed analysis (1) excluding outcomes of interest that occurred in the first 3-y follow-up to reduce the possibility of reverse causality; (2) excluding extreme (1^st^ or 99^th^ quantile) values of exposures (*n*=15,630); (3) further adjusted for C-reactive protein or INFLA-score; (4) excluding individuals with missing covariates included in the fully adjusted model (n=24,646); (5) using propensity score matching as an alternative to the multivariable adjustment to further address residual confounding effect.^48^ This was performed by creating two categories of grip or SMM (in the lowest tertiles or higher level) and using propensity score matching to exclude non-matched individuals based on the set of covariates, applying a 1:1 ratio and a caliper of 0.2.

Analyses were conducted using R 4.1.1. We corrected for multiple comparisons with false discovery rate (FDR) control given potentially inflated type I errors due to multiple tests. Two-sided FDR-adjusted P value (Q value) < 0.05 was considered significant.

## Supplemental information

Document S1. Figure S1 and Tables S2‒S14 and S16
