# Supplementary material for: Risk of subsequent gastrointestinal disease assessed by skeletal muscle strength and mass in a prospective cohort study
Source: iScience. 2024 Mar 5;27(4):109341. doi: 10.1016/j.isci.2024.109341 (PMC10972794; doi:10.1016/j.isci.2024.109341)
Supplement: Document S1. Figure S1 and Tables S2‒S14 and S16 [file mmc1.pdf]

iScience, Volume *27*

Supplemental information

Risk of subsequent gastrointestinal disease assessed by skeletal muscle strength and mass in a prospective cohort study

Lintao Dan, Pei Qin, Siyuan Xie, Yuhao Sun, Tian Fu, Xixian Ruan, Wenming Shi, Jie Chen, Jianting Cai, and Xue Li

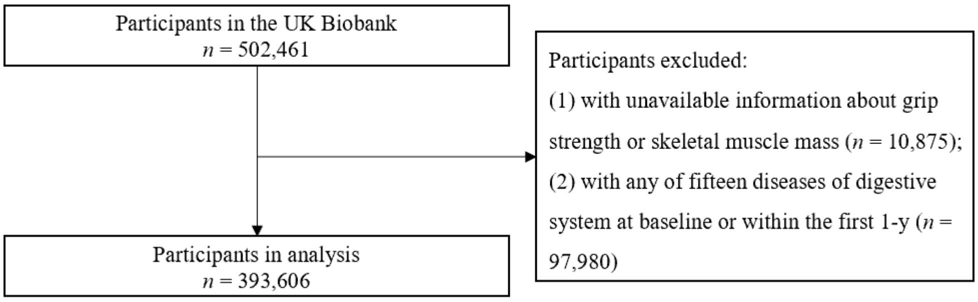

Figure S1. Flowchart of inclusion of study participants

Table S1. Primary analysis for associations of grip strength and skeletal muscle mass and risk of twenty-four gastrointestinal diseases

See supplementary file uploaded as Excel file

CI, confidence interval; HR, hazard ratio

Table S2 The proportion of disease incidence attributable to having the lowest tertiles of grip strength

Diseases	Population attributable fractions (%)<br>Attributable to the lowest tertile of grip strength (95% CI)
Barrett's esophagus	7.0 (4.4, 9.6)
Gastroesophageal reflux disease	5.1 (4.3, 6.0)
Gastritis and duodenitis	6.7 (5.7, 7.7)
Celiac disease	9.4 (5.2, 13.6)
Crohn's disease	5.3 (0.0, 10.5)
Ulcerative colitis	3.9 (0.1, 7.8)
Intestinal diverticular disease	3.4 (2.6, 4.2)
Irritable bowel syndrome	10.5 (8.6, 12.4)
Peptic ulcer	10.9 (8.7, 13.1)
Acute pancreatitis	5.8 (1.6, 9.9)
Chronic pancreatitis	13.7 (5.4, 22.0)
Cholangitis	7.5 (1.8, 13.2)
Cholelithiasis	4.4 (2.9, 5.9)
Non-alcoholic fatty liver disease	10.2 (7.8, 12.5)
Liver cirrhosis	13.8 (10.0, 17.6)
Liver cancers	15.9 (7.0, 24.9)

CI, confidence interval.

Table S3 The proportion of disease incidence attributable to having the lowest tertiles of skeletal muscle mass

Diseases	Population attributable fractions (%)
	Attributable to the lowest tertile of skeletal muscle mass (95% CI)
Barrett's esophagus	7.1 (4.4, 9.7)
Gastroesophageal reflux disease	9.8 (8.9, 10.6)
Esophageal cancer	6.8 (1.1, 12.6)
Gastritis and duodenitis	5.5 (4.5, 6.4)
Intestinal diverticular disease	4.5 (2.7, 6.4)
Irritable bowel syndrome	2.7 (1.0, 4.5)
Peptic ulcer	9.9 (7.7, 12.1)
Small intestinal cancer	14.3 (3.5, 25.0)
Colorectal cancer	4.0 (1.7, 6.3)
Acute pancreatitis	23.6 (19.5, 27.6)
Chronic pancreatitis	13.0 (4.4, 21.5)
Cholangitis	16.8 (11.1, 22.5)
Cholecystitis	27.6 (24.3, 30.8)
Cholelithiasis	27.5 (26.1, 29.0)
Gallbladder and biliary cancer	11.2 (3.9, 18.4)
Non-alcoholic fatty liver disease	38.2 (36.0, 40.4)
Liver cirrhosis	17.7 (14.0, 21.5)
Liver cancers	21.5 (12.7, 30.2)

CI, confidence interval.

Table S4 Associations between skeletal muscle mass in 1-SD increment and risk of twenty-four gastrointestinal diseases when applying different method to address impact of body size

	adjust using whole body fat mass		adjust using BMI	
	HR (95% CI)[1]	Q [2]	HR (95% CI)	Q
**Esophagus**				
**Barrett's esophagus**	**0.85 (0.82, 0.89)**	**<0.001**	**0.83 (0.80, 0.86)**	**<0.001**
**Gastroesophageal reflux disease**	**0.81 (0.80, 0.82)**	**<0.001**	**0.82 (0.81, 0.83)**	**<0.001**
**Esophageal cancer**	**0.91 (0.84, 0.98)**	**0.015**	0.94 (0.87, 1.00)	0.109
**Stomach and bowel**				
**Gastritis and duodenitis**	**0.92 (0.91, 0.94)**	**<0.001**	**0.89 (0.87, 0.90)**	**<0.001**
**Celiac disease**	**1.22 (1.17, 1.28)**	**<0.001**	**1.22 (1.16, 1.29)**	**<0.001**
**Crohn's disease**	0.97 (0.90, 1.04)	0.399	0.94 (0.87, 1.01)	0.167
**Intestinal diverticular disease**	1.00 (0.95, 1.05)	0.922	1.02 (0.97, 1.07)	0.591
**Ulcerative colitis**	**0.83 (0.82, 0.84)**	**<0.001**	**0.87 (0.86, 0.88)**	**<0.001**
**Irritable bowel syndrome**	**0.96 (0.94, 0.99)**	**0.006**	**0.95 (0.92, 0.97)**	**<0.001**
**Peptic ulcer**	**0.87 (0.85, 0.90)**	**<0.001**	**0.85 (0.82, 0.87)**	**<0.001**
**Gastric cancer**	**0.91 (0.84, 0.99)**	**0.036**	0.93 (0.86, 1.01)	0.125
**Small intestinal cancer**	**0.83 (0.71, 0.96)**	**0.021**	0.94 (0.82, 1.08)	0.517
**Colorectal cancer**	**0.93 (0.90, 0.96)**	**<0.001**	0.99 (0.96, 1.02)	0.517
**Pancreas**				
**Acute pancreatitis**	**0.63 (0.58, 0.67)**	**<0.001**	**0.77 (0.73, 0.82)**	**<0.001**
**Chronic pancreatitis**	0.90 (0.80, 1.01)	0.08	0.94 (0.84, 1.04)	0.329
**Pancreatic cancer**	**0.88 (0.83, 0.95)**	**0.001**	1.01 (0.95, 1.08)	0.682
**Gallbladder and biliary**				
**Cholangitis**	**0.72 (0.66, 0.79)**	**<0.001**	**0.84 (0.78, 0.90)**	**<0.001**
**Cholecystitis**	**0.58 (0.55, 0.62)**	**<0.001**	**0.71 (0.68, 0.74)**	**<0.001**
**Cholelithiasis**	**0.54 (0.53, 0.56)**	**<0.001**	**0.69 (0.68, 0.70)**	**<0.001**
**Gallbladder and biliary cancer**	**0.83 (0.75, 0.92)**	**0.001**	0.93 (0.84, 1.02)	0.168
**Liver diseases**				
**Non-alcoholic fatty liver disease**	**0.38 (0.37, 0.40)**	**<0.001**	**0.57 (0.55, 0.59)**	**<0.001**
**Liver cirrhosis**	**0.74 (0.70, 0.79)**	**<0.001**	**0.79 (0.76, 0.83)**	**<0.001**
**Liver cancers**	**0.81 (0.71, 0.92)**	**0.003**	**0.84 (0.75, 0.94)**	**0.004**
**Appendix diseases**				
**Appendicitis**	0.98 (0.93, 1.02)	0.317	1.00 (0.95, 1.04)	0.851

CI, confidence interval; HR, hazard ratio

[1] adjusted for age, sex, ethnicity, Townsend deprivation index, education, physical activity, BMI, healthy diet, alcohol consumption, physical activity, BMI, smoking status and Charlson comorbidity index.

[2] HR (95% CI) in bold indicate significant association after multiple testing.

Table S5 Subgroup analyses for the associations between grip strength in 1-SD increment and risk of twenty-four gastrointestinal diseases stratified by age and sex [1,2]

	Age			Sex		
	<60<br>HR (95% CI)<br>(*n* = 233,226)	≥60<br>HR (95% CI)<br>(*n* = 160,380)	*P*-interaction	Male<br>HR (95% CI)<br>(*n* =181,622)	Female<br>HR (95% CI)<br>(*n* = 211,984)	*P*-interaction
Barrett's esophagus	**0.85 (0.81, 0.89)**	**0.89 (0.85, 0.93)**	0.17	**0.92 (0.88, 0.96)**	**0.88 (0.83, 0.93)**	0.22
Gastroesophageal reflux disease	**0.89 (0.87, 0.90)**	**0.90 (0.88, 0.91)**	0.17	**0.95 (0.93, 0.97)**	**0.88 (0.86, 0.89)**	**<0.001**
Esophageal cancer	**0.84 (0.75, 0.93)**	1.00 (0.92, 1.09)	**0.046**	0.98 (0.91, 1.06)	1.00 (0.88, 1.14)	0.94
Gastritis and duodenitis	**0.85 (0.84, 0.87)**	**0.89 (0.87, 0.90)**	**0.046**	**0.91 (0.89, 0.93)**	**0.87 (0.86, 0.89)**	0.11
Celiac disease	**0.88 (0.82, 0.95)**	**0.84 (0.77, 0.92)**	0.40	**0.86 (0.78, 0.94)**	**0.88 (0.82, 0.95)**	0.80
Crohn's disease	0.98 (0.90, 1.07)	**0.84 (0.75, 0.95)**	0.16	0.95 (0.85, 1.06)	**0.88 (0.80, 0.98)**	0.80
Ulcerative colitis	0.94 (0.88, 1.00)	0.97 (0.89, 1.05)	0.57	0.95 (0.89, 1.02)	0.94 (0.87, 1.01)	0.98
Intestinal diverticular disease	**0.89 (0.88, 0.91)**	**0.92 (0.91, 0.94)**	0.15	**0.96 (0.94, 0.97)**	**0.93 (0.92, 0.94)**	**<0.001**
Irritable bowel syndrome	**0.84 (0.81, 0.86)**	**0.86 (0.83, 0.90)**	0.17	**0.92 (0.88, 0.97)**	**0.81 (0.78, 0.83)**	**<0.001**
Peptic ulcer	**0.80 (0.77, 0.83)**	**0.84 (0.81, 0.87)**	0.16	**0.87 (0.84, 0.90)**	**0.81 (0.78, 0.85)**	0.24
Gastric cancer	0.90 (0.80, 1.02)	1.04 (0.95, 1.14)	0.17	1.05 (0.96, 1.15)	0.98 (0.85, 1.13)	0.80
Small intestinal cancer	0.89 (0.73, 1.09)	0.94 (0.79, 1.11)	0.71	1.09 (0.91, 1.31)	0.84 (0.69, 1.01)	0.26
Colorectal cancer	**0.94 (0.90, 0.98)**	0.97 (0.93, 1.00)	0.40	0.99 (0.95, 1.03)	1.03 (0.99, 1.08)	0.14
Acute pancreatitis	**0.86 (0.80, 0.92)**	**0.91 (0.85, 0.99)**	0.34	**0.90 (0.84, 0.97)**	**0.91 (0.85, 0.98)**	0.86
Chronic pancreatitis	**0.78 (0.68, 0.91)**	0.90 (0.79, 1.04)	0.22	0.88 (0.78, 1.00)	0.83 (0.70, 0.99)	0.80

Pancreatic cancer	**0.91 (0.83, 1.01)**	0.97 (0.90, 1.05)	0.40	1.01 (0.93, 1.10)	1.00 (0.91, 1.10)	0.80
Cholangitis	**0.85 (0.76, 0.95)**	**0.90 (0.82, 0.98)**	0.39	0.99 (0.90, 1.08)	**0.83 (0.74, 0.92)**	0.22
Cholecystitis	**0.92 (0.87, 0.97)**	1.01 (0.95, 1.07)	0.17	1.03 (0.97, 1.10)	**0.93 (0.88, 0.99)**	0.80
Cholelithiasis	**0.91 (0.89, 0.93)**	**0.93 (0.91, 0.96)**	0.17	**0.96 (0.93, 0.99)**	**0.91 (0.89, 0.94)**	0.80
Gallbladder and biliary cancer	0.87 (0.75, 1.01)	0.94 (0.84, 1.05)	0.40	0.96 (0.84, 1.10)	0.98 (0.86, 1.11)	0.80
Non-alcoholic fatty liver disease	**0.84 (0.81, 0.87)**	**0.88 (0.84, 0.92)**	0.17	0.88 (0.84, 0.92)	**0.83 (0.79, 0.86)**	**0.030**
Liver cirrhosis	**0.74 (0.69, 0.79)**	**0.85 (0.80, 0.91)**	**0.043**	0.81 (0.77, 0.86)	**0.81 (0.75, 0.88)**	0.94
Liver cancers	**0.72 (0.61, 0.84)**	**0.79 (0.70, 0.91)**	0.34	**0.81 (0.71, 0.92)**	**0.77 (0.64, 0.93)**	0.80
Appendicitis	1.04 (0.98, 1.10)	1.01 (0.93, 1.10)	0.71	1.03 (0.96, 1.10)	0.99 (0.92, 1.05)	0.80

CI, confidence interval; HR, hazard ratio

[1] HR (95% CI) in bold indicate significant association after multiple testing.

[2] *P*-interaction had been corrected for multiple comparisons with FDR control.

Table S6. Subgroup analyses for the associations between grip strength in 1-SD increment and risk of twenty-four gastrointestinal diseases stratified by alcohol consumption, smoking status, and diet [1, 2]

	**Alcohol consumption**			**Smoking status**			**Diet**		
	None to moderate consumption (*n* = 81,337)	Heavy consumption (*n* = 312,269)	*P*-interaction	Never smoked (*n* = 221,513)	Previous or current smokers (*n* = 172,093)	*P*-interaction	Unhealthy diet (*n* = 105,392)	Healthy diet (*n* = 288,214)	*P*-interaction
Barrett's esophagus	**0.90 (0.84, 0.96)**	**0.91 (0.87, 0.94)**	0.99	**0.88 (0.84, 0.93)**	**0.92 (0.88, 0.97)**	0.48	**0.94 (0.88, 0.99)**	**0.89 (0.85, 0.93)**	0.69
Gastroesophageal reflux disease	**0.92 (0.90, 0.95)**	**0.91 (0.89, 0.92)**	0.44	**0.90 (0.89, 0.92)**	**0.91 (0.90, 0.93)**	0.68	**0.94 (0.92, 0.96)**	**0.90 (0.88, 0.91)**	<0.001
Esophageal cancer	1.01 (0.89, 1.15)	0.98 (0.91, 1.06)	0.99	1.03 (0.92, 1.15)	0.97 (0.89, 1.05)	0.62	1.01 (0.91, 1.13)	0.98 (0.90, 1.06)	0.92
Gastritis and duodenitis	**0.89 (0.86, 0.92)**	**0.89 (0.88, 0.90)**	0.99	**0.89 (0.87, 0.90)**	**0.89 (0.88, 0.91)**	0.82	**0.90 (0.88, 0.92)**	**0.89 (0.87, 0.90)**	0.66
Celiac disease	**0.84 (0.72, 0.96)**	**0.88 (0.83, 0.93)**	0.89	**0.88 (0.82, 0.95)**	**0.86 (0.79, 0.94)**	0.62	**0.89 (0.81, 0.99)**	**0.86 (0.81, 0.92)**	0.92
Crohn's disease	0.93 (0.79, 1.10)	**0.91 (0.84, 0.98)**	0.99	0.95 (0.85, 1.05)	**0.89 (0.81, 0.98)**	0.68	0.90 (0.79, 1.02)	0.92 (0.84, 1.00)	0.92
Ulcerative colitis	0.92 (0.83, 1.03)	0.95 (0.90, 1.01)	0.99	**0.90 (0.84, 0.98)**	0.98 (0.91, 1.05)	0.38	**0.89 (0.82, 0.98)**	0.97 (0.91, 1.03)	0.47
Intestinal diverticular disease	**0.95 (0.93, 0.97)**	**0.94 (0.93, 0.95)**	0.25	**0.93 (0.91, 0.94)**	**0.95 (0.94, 0.97)**	0.05	**0.95 (0.94, 0.97)**	**0.94 (0.92, 0.95)**	0.051

Irritable bowel syndrome	**0.84 (0.78, 0.89)**	**0.84 (0.81, 0.86)**	0.99	**0.82 (0.79, 0.85)**	**0.85 (0.82, 0.89)**	0.34	**0.84 (0.80, 0.88)**	**0.84 (0.81, 0.86)**	0.92
Peptic ulcer	**0.83 (0.78, 0.88)**	**0.85 (0.82, 0.87)**	0.99	**0.82 (0.79, 0.85)**	**0.86 (0.83, 0.90)**	0.34	**0.85 (0.81, 0.89)**	**0.84 (0.82, 0.87)**	0.92
Gastric cancer	1.03 (0.88, 1.21)	1.04 (0.95, 1.13)	0.99	1.03 (0.92, 1.16)	1.03 (0.94, 1.14)	0.75	1.03 (0.90, 1.17)	1.04 (0.94, 1.14)	0.92
Small intestinal cancer	1.21 (0.92, 1.61)	0.90 (0.78, 1.05)	0.42	0.89 (0.74, 1.07)	1.05 (0.87, 1.26)	0.62	0.86 (0.66, 1.11)	1.00 (0.86, 1.17)	0.72
Colorectal cancer	0.96 (0.91, 1.02)	1.03 (0.99, 1.06)	0.25	1.00 (0.95, 1.04)	1.02 (0.98, 1.07)	0.74	1.00 (0.95, 1.05)	1.02 (0.98, 1.05)	0.92
Acute pancreatitis	**0.79 (0.70, 0.89)**	**0.94 (0.89, 0.99)**	0.41	0.97 (0.90, 1.05)	**0.85 (0.80, 0.92)**	0.34	**0.85 (0.78, 0.93)**	0.94 (0.89, 1.00)	0.27
Chronic pancreatitis	0.95 (0.77, 1.16)	**0.84 (0.75, 0.95)**	0.42	0.84 (0.71, 0.99)	0.88 (0.77, 1.01)	0.62	**0.79 (0.67, 0.93)**	0.92 (0.80, 1.05)	0.66
Pancreatic cancer	1.02 (0.90, 1.16)	1.00 (0.94, 1.08)	0.99	1.08 (0.98, 1.18)	0.95 (0.87, 1.03)	0.36	1.02 (0.90, 1.15)	1.01 (0.94, 1.08)	0.98
Cholangitis	0.95 (0.81, 1.11)	**0.91 (0.84, 0.98)**	0.99	**0.89 (0.80, 0.98)**	0.94 (0.86, 1.03)	0.75	0.96 (0.84, 1.08)	**0.90 (0.83, 0.98)**	0.84
Cholecystitis	1.04 (0.93, 1.15)	0.97 (0.92, 1.01)	0.99	0.98 (0.92, 1.04)	0.98 (0.92, 1.04)	>0.99	0.95 (0.88, 1.03)	0.99 (0.94, 1.04)	0.66
Cholelithiasis	0.95 (0.90, 1.00)	**0.93 (0.91, 0.95)**	0.99	**0.95 (0.92, 0.98)**	**0.92 (0.89, 0.94)**	0.13	**0.92 (0.89, 0.95)**	**0.94 (0.92, 0.96)**	0.57
Gallbladder and biliary cancer	0.96 (0.78, 1.17)	0.98 (0.88, 1.08)	0.99	1.02 (0.89, 1.17)	0.93 (0.82, 1.05)	0.62	1.02 (0.86, 1.20)	0.95 (0.85, 1.06)	0.98

Non-alcoholic fatty liver disease	**0.88 (0.83, 0.94)**	**0.85 (0.82, 0.88)**	0.44	**0.85 (0.81, 0.89)**	**0.85 (0.82, 0.89)**	0.82	**0.86 (0.82, 0.91)**	**0.85 (0.82, 0.88)**	0.92
Liver cirrhosis	**0.79 (0.73, 0.85)**	**0.82 (0.78, 0.87)**	0.99	**0.79 (0.74, 0.85)**	**0.82 (0.77, 0.87)**	0.62	**0.82 (0.76, 0.89)**	**0.80 (0.76, 0.85)**	0.92
Liver cancers	0.83 (0.68, 1.02)	**0.78 (0.69, 0.89)**	0.99	**0.80 (0.68, 0.95)**	**0.79 (0.69, 0.91)**	0.82	**0.81 (0.69, 0.96)**	**0.79 (0.69, 0.90)**	0.98
Appendicitis	1.02 (0.92, 1.12)	1.00 (0.95, 1.06)	0.99	0.99 (0.93, 1.05)	1.03 (0.96, 1.11)	0.62	0.97 (0.89, 1.05)	1.02 (0.97, 1.08)	0.66

CI, confidence interval; HR, hazard ratio

[1] HR (95% CI) in bold indicate significant association after multiple testing.

[2] *P*-interaction had been corrected for multiple comparisons with FDR control.

Table S7. Subgroup analyses for the associations between skeletal muscle mass in 1-SD increment and risk of twenty-four gastrointestinal diseases stratified by age and sex [1, 2].

	Age			Sex		
	<60<br>HR (95% CI)<br>(*n* = 233,226)	≥60<br>HR (95% CI)<br>(*n* = 160,380)	*P*-interaction	Male<br>HR (95% CI)<br>(*n* =181,622)	Female<br>HR (95% CI)<br>(*n* = 211,984)	*P*-interaction
Barrett's esophagus	**0.82 (0.78, 0.86)**	**0.85 (0.81, 0.89)**	0.22	**0.90 (0.86, 0.94)**	**0.82 (0.78, 0.87)**	**0.016**
Gastroesophageal reflux disease	**0.77 (0.76, 0.78)**	**0.86 (0.85, 0.88)**	**<0.001**	**0.91 (0.89, 0.93)**	**0.76 (0.75, 0.78)**	**<0.001**
Esophageal cancer	**0.71 (0.64, 0.80)**	0.94 (0.86, 1.03)	**<0.001**	**0.85 (0.78, 0.92)**	1.06 (0.93, 1.20)	**0.018**
Gastritis and duodenitis	**0.87 (0.86, 0.89)**	**0.92 (0.90, 0.94)**	**<0.001**	**0.94 (0.92, 0.96)**	**0.90 (0.88, 0.92)**	**0.045**
Celiac disease	**1.16 (1.08, 1.24)**	**1.40 (1.29, 1.53)**	**<0.001**	**1.29 (1.23, 1.35)**	**1.19 (1.12, 1.28)**	0.20
Crohn's disease	0.94 (0.86, 1.03)	1.01 (0.89, 1.14)	0.61	0.95 (0.85, 1.06)	0.96 (0.87, 1.06)	0.93
Ulcerative colitis	1.02 (0.96, 1.09)	1.02 (0.94, 1.10)	0.79	1.03 (0.96, 1.11)	1.00 (0.93, 1.08)	0.89
Intestinal diverticular disease	**0.75 (0.74, 0.76)**	**0.86 (0.85, 0.88)**	**<0.001**	**0.84 (0.83, 0.86)**	**0.83 (0.81, 0.84)**	**<0.001**
Irritable bowel syndrome	**0.93 (0.90, 0.96)**	1.03 (0.98, 1.07)	**<0.001**	0.99 (0.94, 1.05)	**0.94 (0.92, 0.97)**	**0.009**
Peptic ulcer	**0.84 (0.81, 0.87)**	**0.82 (0.79, 0.86)**	0.90	**0.90 (0.86, 0.93)**	**0.83 (0.80, 0.87)**	**0.049**
Gastric cancer	**0.79 (0.70, 0.90)**	0.95 (0.86, 1.04)	**0.04**	0.96 (0.87, 1.06)	0.89 (0.78, 1.03)	0.86
Small intestinal cancer	**0.69 (0.56, 0.85)**	0.85 (0.71, 1.02)	0.16	**0.79 (0.65, 0.97)**	0.83 (0.69, 1.01)	0.59
Colorectal cancer	**0.83 (0.79, 0.87)**	**0.92 (0.88, 0.95)**	**<0.001**	**0.89 (0.86, 0.93)**	0.97 (0.93, 1.01)	**0.003**
Acute pancreatitis	**0.62 (0.58, 0.67)**	**0.70 (0.64, 0.75)**	0.050	**0.72 (0.67, 0.78)**	**0.63 (0.58, 0.68)**	0.06
Chronic pancreatitis	**0.82 (0.70, 0.96)**	0.88 (0.76, 1.02)	0.33	0.91 (0.79, 1.05)	0.84 (0.70, 1.00)	0.40

Pancreatic cancer	**0.75 (0.68, 0.83)**	0.92 (0.85, 0.99)	**0.005**	0.93 (0.85, 1.02)	**0.88 (0.80, 0.96)**	0.35
Cholangitis	**0.67 (0.59, 0.75)**	**0.76 (0.69, 0.84)**	0.09	**0.80 (0.72, 0.88)**	**0.71 (0.63, 0.79)**	0.37
Cholecystitis	**0.60 (0.56, 0.63)**	**0.67 (0.62, 0.71)**	0.06	**0.69 (0.65, 0.74)**	**0.59 (0.56, 0.63)**	**0.049**
Cholelithiasis	**0.57 (0.56, 0.59)**	**0.67 (0.65, 0.69)**	**<0.001**	**0.70 (0.68, 0.73)**	**0.58 (0.56, 0.59)**	**<0.001**
Gallbladder and biliary cancer	**0.75 (0.64, 0.87)**	**0.82 (0.73, 0.92)**	0.42	0.92 (0.80, 1.06)	**0.76 (0.67, 0.87)**	0.21
Non-alcoholic fatty liver disease	**0.50 (0.48, 0.52)**	**0.52 (0.49, 0.55)**	0.16	**0.55 (0.52, 0.58)**	**0.46 (0.44, 0.48)**	**<0.001**
Liver cirrhosis	**0.75 (0.70, 0.80)**	**0.70 (0.65, 0.75)**	0.60	**0.73 (0.68, 0.78)**	**0.78 (0.72, 0.84)**	0.58
Liver cancers	**0.77 (0.64, 0.92)**	**0.71 (0.61, 0.82)**	0.79	**0.70 (0.60, 0.81)**	0.94 (0.78, 1.13)	**0.003**
Appendicitis	1.01 (0.95, 1.07)	0.95 (0.87, 1.03)	0.38	0.98 (0.91, 1.05)	0.96 (0.90, 1.02)	0.89

CI, confidence interval; HR, hazard ratio

[1] HR (95% CI) in bold indicate significant association after multiple testing.

[2] *P*-interaction had been corrected for multiple comparisons with FDR control.

Table S8. Subgroup analyses for the associations between skeletal muscle mass in 1-SD increment and risk of twenty-four gastrointestinal diseases stratified by alcohol consumption, smoking status, and diet[1, 2]

	**Alcohol consumption**			**Smoking status**			**Diet**		
	None to moderate consumption (*n* = 81,337)	Heavy consumption (*n* = 312,269)	*P*-interaction	Never smoked (*n* = 221,513)	Previous or current smokers (*n* = 172,093)	*P*-interaction	Unhealthy diet (*n* = 105,392)	Healthy diet (*n* = 288,214)	*P*-interaction
Barrett's esophagus	**0.89 (0.82, 0.95)**	**0.87 (0.83, 0.90)**	0.79	**0.88 (0.83, 0.92)**	**0.87 (0.83, 0.91)**	0.98	**0.87 (0.82, 0.93)**	**0.87 (0.84, 0.91)**	0.97
Gastroesophageal reflux disease	**0.87 (0.84, 0.89)**	**0.81 (0.80, 0.83)**	**0.001**	**0.81 (0.79, 0.82)**	**0.84 (0.83, 0.86)**	**0.014**	**0.85 (0.83, 0.87)**	**0.81 (0.80, 0.82)**	**<0.001**
Esophageal cancer	0.91 (0.79, 1.04)	**0.91 (0.84, 0.98)**	0.87	0.90 (0.80, 1.02)	0.91 (0.84, 0.99)	0.98	0.90 (0.80, 1.01)	0.91 (0.84, 1.00)	0.97
Gastritis and duodenitis	**0.94 (0.91, 0.97)**	**0.91 (0.90, 0.92)**	0.26	**0.90 (0.88, 0.91)**	**0.93 (0.92, 0.95)**	**0.022**	**0.94 (0.92, 0.97)**	**0.90 (0.89, 0.92)**	0.05
Celiac disease	**1.32 (1.14, 1.52)**	**1.24 (1.18, 1.30)**	0.98	**1.26 (1.19, 1.33)**	**1.24 (1.13, 1.35)**	0.70	**1.32 (1.20, 1.46)**	**1.23 (1.17, 1.30)**	0.97
Crohn's disease	1.00 (0.84, 1.18)	0.94 (0.87, 1.02)	0.79	0.97 (0.88, 1.08)	0.93 (0.85, 1.03)	0.83	1.01 (0.89, 1.15)	0.93 (0.85, 1.02)	0.97
Ulcerative colitis	1.05 (0.94, 1.18)	1.01 (0.95, 1.07)	0.85	1.05 (0.97, 1.13)	0.99 (0.92, 1.06)	0.72	1.06 (0.97, 1.16)	0.99 (0.93, 1.06)	0.97
Intestinal diverticular disease	**0.85 (0.83, 0.87)**	**0.84 (0.83, 0.85)**	0.06	**0.81 (0.80, 0.83)**	**0.86 (0.85, 0.87)**	**<0.001**	**0.85 (0.83, 0.87)**	**0.83 (0.82, 0.84)**	**0.019**

Irritable bowel syndrome	0.95 (0.89, 1.01)	**0.96 (0.94, 0.99)**	0.79	**0.96 (0.93, 1.00)**	**0.95 (0.92, 0.99)**	0.83	0.96 (0.91, 1.01)	**0.96 (0.93, 0.99)**	0.97
Peptic ulcer	**0.89 (0.84, 0.95)**	**0.85 (0.83, 0.88)**	0.75	**0.83 (0.80, 0.87)**	**0.89 (0.85, 0.92)**	0.15	**0.89 (0.84, 0.94)**	**0.85 (0.82, 0.88)**	0.49
Gastric cancer	0.84 (0.71, 1.00)	0.96 (0.88, 1.05)	0.26	0.91 (0.80, 1.02)	0.95 (0.86, 1.06)	0.72	0.95 (0.83, 1.09)	0.92 (0.84, 1.02)	0.97
Small intestinal cancer	0.76 (0.56, 1.03)	0.83 (0.71, 0.97)	0.84	0.82 (0.68, 1.00)	0.81 (0.66, 0.99)	0.83	0.81 (0.61, 1.06)	0.81 (0.69, 0.95)	0.97
Colorectal cancer	**0.93 (0.88, 0.99)**	**0.93 (0.89, 0.96)**	0.79	**0.93 (0.89, 0.97)**	**0.92 (0.89, 0.96)**	0.83	**0.92 (0.87, 0.98)**	**0.92 (0.89, 0.96)**	0.97
Acute pancreatitis	**0.74 (0.65, 0.84)**	**0.65 (0.61, 0.69)**	0.14	**0.64 (0.60, 0.70)**	**0.69 (0.64, 0.75)**	0.25	**0.67 (0.61, 0.74)**	**0.67 (0.62, 0.71)**	0.97
Chronic pancreatitis	1.07 (0.86, 1.33)	**0.83 (0.73, 0.94)**	0.05	**0.80 (0.67, 0.95)**	0.94 (0.82, 1.08)	0.25	**0.77 (0.64, 0.92)**	0.96 (0.84, 1.10)	0.22
Pancreatic cancer	0.89 (0.78, 1.02)	**0.91 (0.84, 0.97)**	0.87	**0.88 (0.80, 0.96)**	0.92 (0.84, 1.00)	0.80	0.91 (0.81, 1.04)	**0.90 (0.84, 0.97)**	0.97
Cholangitis	**0.77 (0.65, 0.92)**	**0.74 (0.68, 0.81)**	0.79	**0.77 (0.69, 0.86)**	**0.73 (0.66, 0.81)**	0.72	**0.76 (0.66, 0.87)**	**0.74 (0.68, 0.81)**	0.97
Cholecystitis	**0.73 (0.65, 0.81)**	**0.61 (0.58, 0.64)**	0.22	**0.59 (0.55, 0.63)**	**0.67 (0.63, 0.71)**	**0.046**	**0.66 (0.61, 0.72)**	**0.61 (0.58, 0.65)**	0.91
Cholelithiasis	**0.66 (0.63, 0.70)**	**0.60 (0.59, 0.62)**	0.14	**0.59 (0.57, 0.60)**	**0.64 (0.62, 0.66)**	**0.004**	**0.65 (0.63, 0.68)**	**0.60 (0.58, 0.61)**	**0.013**
Gallbladder and biliary cancer	0.80 (0.64, 1.00)	**0.84 (0.76, 0.94)**	0.85	**0.84 (0.73, 0.96)**	**0.83 (0.73, 0.95)**	0.98	0.86 (0.72, 1.03)	**0.82 (0.73, 0.92)**	0.97

Non-alcoholic fatty liver disease	**0.56 (0.52, 0.60)**	**0.49 (0.47, 0.51)**	**0.001**	**0.49 (0.46, 0.51)**	**0.53 (0.50, 0.55)**	**0.022**	**0.53 (0.50, 0.56)**	**0.49 (0.48, 0.52)**	0.22
Liver cirrhosis	**0.76 (0.70, 0.84)**	**0.74 (0.70, 0.79)**	0.42	**0.70 (0.65, 0.75)**	**0.79 (0.74, 0.84)**	**0.022**	**0.78 (0.72, 0.85)**	**0.74 (0.69, 0.78)**	0.93
Liver cancers	**0.66 (0.52, 0.83)**	**0.81 (0.71, 0.93)**	0.06	**0.75 (0.63, 0.90)**	**0.78 (0.67, 0.90)**	0.98	**0.70 (0.58, 0.85)**	**0.81 (0.70, 0.94)**	0.32
Appendicitis	0.94 (0.85, 1.05)	0.97 (0.92, 1.03)	0.77	0.97 (0.91, 1.03)	0.96 (0.90, 1.03)	0.98	0.97 (0.89, 1.06)	0.96 (0.91, 1.02)	0.97

CI, confidence interval; HR, hazard ratio

[1] HR (95% CI) in bold indicate significant association after multiple testing.

[2] *P*-interaction had been corrected for multiple comparisons with FDR control.

Table S9. Sensitivity analysis for associations of grip strength and skeletal muscle mass and risk of twenty-four gastrointestinal diseases excluding incident cases in the first 3-y follow-up (n = 19,037)[1, 2]

	Grip strength		Skeletal muscle mass	
	HR (95% CI)	*Q*	HR (95% CI)	*Q*
**Esophagus**				
Barrett's esophagus	**0.90 (0.87, 0.93)**	**<0.001**	**0.86 (0.83, 0.89)**	**<0.001**
Gastroesophageal reflux disease	**0.91 (0.90, 0.92)**	**<0.001**	**0.83 (0.82, 0.84)**	**<0.001**
Esophageal cancer	1.00 (0.93, 1.07)	0.92	**0.88 (0.81, 0.95)**	**0.001**
**Stomach and bowel**				
Gastritis and duodenitis	**0.90 (0.89, 0.91)**	**<0.001**	**0.91 (0.90, 0.93)**	**<0.001**
Celiac disease	**0.90 (0.84, 0.95)**	**0.002**	**1.23 (1.17, 1.30)**	**<0.001**
Crohn's disease	0.93 (0.86, 1.01)	0.16	0.93 (0.86, 1.02)	0.14
Ulcerative colitis	0.95 (0.90, 1.01)	0.16	1.01 (0.95, 1.07)	0.67
Intestinal diverticular disease	**0.94 (0.93, 0.95)**	**<0.001**	**0.84 (0.83, 0.85)**	**<0.001**
Irritable bowel syndrome	**0.83 (0.81, 0.86)**	**<0.001**	**0.95 (0.92, 0.98)**	**0.001**
Peptic ulcer	**0.85 (0.82, 0.88)**	**<0.001**	**0.85 (0.82, 0.88)**	**<0.001**
Gastric cancer	1.03 (0.95, 1.13)	0.54	0.93 (0.85, 1.02)	0.14
Small intestinal cancer	0.98 (0.85, 1.14)	0.92	**0.77 (0.65, 0.90)**	**0.001**
Colorectal cancer	1.01 (0.97, 1.04)	0.82	**0.91 (0.88, 0.94)**	**<0.001**
**Pancreas**				
Acute pancreatitis	**0.92 (0.87, 0.97)**	**0.009**	**0.67 (0.63, 0.71)**	**<0.001**
Chronic pancreatitis	**0.86 (0.76, 0.97)**	**0.024**	0.93 (0.82, 1.06)	0.29
Pancreatic cancer	1.04 (0.98, 1.12)	0.31	**0.87 (0.82, 0.94)**	**<0.001**
**Gallbladder and biliary**				
Cholangitis	**0.91 (0.84, 0.98)**	**0.022**	**0.74 (0.68, 0.80)**	**<0.001**

Cholecystitis	1.00 (0.96, 1.05)	0.92	**0.63 (0.60, 0.66)**	**<0.001**
Cholelithiasis	**0.94 (0.92, 0.96)**	**<0.001**	**0.61 (0.60, 0.63)**	**<0.001**
Gallbladder and biliary cancer	0.95 (0.86, 1.05)	0.39	**0.82 (0.74, 0.91)**	**<0.001**
**Liver**				
Non-alcoholic fatty liver disease	**0.85 (0.82, 0.88)**	**<0.001**	**0.51 (0.49, 0.53)**	**<0.001**
Chronic liver disease/cirrhosis	**0.81 (0.77, 0.86)**	**<0.001**	**0.74 (0.70, 0.78)**	**<0.001**
Liver cancers	**0.83 (0.73, 0.93)**	**0.004**	**0.74 (0.65, 0.84)**	**<0.001**
**Appendix**				
Appendicitis	0.99 (0.94, 1.05)	0.92	0.99 (0.94, 1.04)	0.62

CI, confidence interval; HR, hazard ratio

[1] adjusted for age, sex, ethnicity, Townsend deprivation index, education, healthy diet, alcohol consumption, physical activity, BMI, smoking status and Charlson comorbidity index.

[2] Two-sided FDR-adjusted P value (Q value) < 0.05 were considered significant.

Table S10. Sensitivity analysis for associations of grip strength and skeletal muscle mass and risk of twenty-four gastrointestinal diseases excluding participants with extreme values (1st or 99th quantile) of exposures (n = 15,630)[1, 2]

	Grip strength		Skeletal muscle mass	
	HR (95% CI)	*Q*	HR (95% CI)	*Q*
**Esophagus**				
Barrett's esophagus	**0.92 (0.89, 0.95)**	**<0.001**	**0.86 (0.83, 0.90)**	**<0.001**
Gastroesophageal reflux disease	**0.90 (0.88, 0.91)**	**<0.001**	**0.82 (0.80, 0.83)**	**<0.001**
Esophageal cancer	0.96 (0.90, 1.04)	0.39	**0.88 (0.82, 0.95)**	**0.002**
**Stomach and bowel**				
Gastritis and duodenitis	**0.89 (0.88, 0.90)**	**<0.001**	**0.91 (0.89, 0.92)**	**<0.001**
Celiac disease	**0.85 (0.80, 0.90)**	**<0.001**	**1.28 (1.21, 1.36)**	**<0.001**
Crohn's disease	**0.86 (0.80, 0.93)**	**<0.001**	0.94 (0.87, 1.01)	0.092
Intestinal diverticular disease	**0.94 (0.93, 0.95)**	**<0.001**	**0.83 (0.82, 0.84)**	**<0.001**
Ulcerative colitis	0.96 (0.91, 1.02)	0.25	1.03 (0.97, 1.08)	0.36
Irritable bowel syndrome	**0.80 (0.78, 0.83)**	**<0.001**	**0.92 (0.89, 0.95)**	**<0.001**
Peptic ulcer	**0.84 (0.82, 0.87)**	**<0.001**	**0.83 (0.81, 0.86)**	**<0.001**
Gastric cancer	1.04 (0.96, 1.12)	0.45	**0.91 (0.84, 0.99)**	**0.039**
Small intestinal cancer	0.97 (0.83, 1.13)	0.73	**0.84 (0.72, 0.98)**	**0.037**
Colorectal cancer	1.02 (0.99, 1.05)	0.32	**0.94 (0.91, 0.97)**	**<0.001**
**Pancreas**				
Acute pancreatitis	**0.92 (0.87, 0.98)**	**0.008**	**0.66 (0.62, 0.70)**	**<0.001**
Chronic pancreatitis	**0.82 (0.73, 0.93)**	**0.003**	**0.86 (0.76, 0.98)**	**0.027**
Pancreatic cancer	0.98 (0.92, 1.05)	0.67	**0.91 (0.85, 0.98)**	**0.011**
**Gallbladder and biliary**				
Cholangitis	**0.87 (0.81, 0.93)**	**<0.001**	**0.69 (0.64, 0.74)**	**<0.001**
Cholecystitis	0.97 (0.92, 1.02)	0.31	**0.61 (0.58, 0.64)**	**<0.001**
Cholelithiasis	**0.93 (0.91, 0.95)**	**<0.001**	**0.60 (0.59, 0.62)**	**<0.001**
Gallbladder and biliary cancer	0.94 (0.85, 1.03)	0.25	**0.85 (0.77, 0.94)**	**0.003**
**Liver diseases**				
Non-alcoholic fatty liver disease	**0.81 (0.78, 0.84)**	**<0.001**	**0.52 (0.50, 0.54)**	**<0.001**
Liver cirrhosis	**0.76 (0.71, 0.80)**	**<0.001**	**0.67 (0.63, 0.72)**	**<0.001**
Liver cancers	**0.82 (0.73, 0.93)**	**0.002**	**0.78 (0.69, 0.88)**	**<0.001**
**Appendix diseases**				
Appendicitis	1.01 (0.96, 1.06)	0.82	0.95 (0.91, 1.00)	0.064

CI, confidence interval; HR, hazard ratio

[1] adjusted for age, sex, ethnicity, Townsend deprivation index, education, healthy diet, alcohol consumption, physical activity, BMI, smoking status and Charlson comorbidity index.

[2] Two-sided FDR-adjusted P value (Q value) < 0.05 were considered significant.

Table S11. Sensitivity analysis for associations of grip strength and skeletal muscle mass (per 1-SD) and risk of twenty-four gastrointestinal diseases further adjusted for C-reactive protein or INFLA-score [1]

	**Further adjusted for C-reactive protein**				**Further adjusted for INFLA-score**			
	**Grip strength**		**Skeletal muscle mass**		**Grip strength**		**Skeletal muscle mass**	
	HR (95% CI)	*Q* [2]	HR (95% CI)	*Q*	HR (95% CI)	*Q* [2]	HR (95% CI)	*Q*
**Esophagus**								
**Barrett's esophagus**	0.90 (0.87, 0.93)	<0.001	0.87 (0.84, 0.90)	<0.001	0.91 (0.88, 0.94)	<0.001	0.89 (0.86, 0.92)	<0.001
**Gastroesophageal reflux disease**	0.91 (0.90, 0.92)	<0.001	0.82 (0.81, 0.83)	<0.001	0.91 (0.90, 0.93)	<0.001	0.84 (0.83, 0.85)	<0.001
**Esophageal cancer**	0.99 (0.92, 1.05)	0.727	0.90 (0.84, 0.97)	0.005	1.00 (0.93, 1.07)	0.978	0.94 (0.88, 1.01)	0.12
**Stomach and bowel**								
**Gastritis and duodenitis**	0.89 (0.88, 0.90)	<0.001	0.91 (0.90, 0.93)	<0.001	0.89 (0.88, 0.91)	<0.001	0.93 (0.92, 0.94)	<0.001
**Celiac disease**	0.87 (0.82, 0.92)	<0.001	1.24 (1.18, 1.30)	<0.001	0.87 (0.83, 0.92)	<0.001	1.27 (1.21, 1.32)	<0.001
**Crohn's disease**	0.91 (0.85, 0.98)	0.02	0.95 (0.88, 1.02)	0.165	0.94 (0.87, 1.01)	0.121	1.07 (0.99, 1.15)	0.113
**Intestinal diverticular disease**	0.94 (0.90, 0.99)	0.037	1.01 (0.96, 1.06)	0.762	0.96 (0.91, 1.01)	0.19	1.09 (1.03, 1.15)	0.003
**Ulcerative colitis**	0.94 (0.93, 0.95)	<0.001	0.84 (0.83, 0.85)	<0.001	0.95 (0.94, 0.96)	<0.001	0.86 (0.85, 0.87)	<0.001
**Irritable bowel syndrome**	0.84 (0.82, 0.86)	<0.001	0.96 (0.94, 0.99)	0.004	0.84 (0.82, 0.86)	<0.001	0.98 (0.95, 1.00)	0.1
**Peptic ulcer**	0.84 (0.82, 0.87)	<0.001	0.86 (0.84, 0.89)	<0.001	0.85 (0.83, 0.88)	<0.001	0.89 (0.86, 0.91)	<0.001
**Gastric cancer**	1.03 (0.96, 1.11)	0.558	0.93 (0.86, 1.01)	0.091	1.04 (0.97, 1.13)	0.378	0.96 (0.89, 1.04)	0.376
**Small intestinal cancer**	0.97 (0.85, 1.10)	0.693	0.82 (0.71, 0.94)	0.005	0.98 (0.86, 1.11)	0.812	0.85 (0.74, 0.97)	0.033
**Colorectal cancer**	1.01 (0.98, 1.04)	0.693	0.92 (0.89, 0.95)	<0.001	1.01 (0.98, 1.04)	0.463	0.94 (0.91, 0.97)	<0.001
**Pancreas**								
**Acute pancreatitis**	0.91 (0.86, 0.96)	0.001	0.66 (0.63, 0.70)	<0.001	0.92 (0.88, 0.97)	0.005	0.70 (0.66, 0.74)	<0.001

**Chronic pancreatitis**	0.86 (0.78, 0.95)	0.009	0.88 (0.79, 0.97)	0.02	0.88 (0.79, 0.97)	0.024	0.93 (0.83, 1.03)	0.197
**Pancreatic cancer**	1.01 (0.95, 1.07)	0.803	0.90 (0.85, 0.96)	0.003	1.02 (0.96, 1.08)	0.757	0.93 (0.87, 0.99)	0.042
**Gallbladder and biliary**								
**Cholangitis**	0.91 (0.85, 0.98)	0.02	0.75 (0.69, 0.81)	<0.001	0.93 (0.87, 1.00)	0.066	0.78 (0.73, 0.85)	<0.001
**Cholecystitis**	0.98 (0.94, 1.02)	0.401	0.63 (0.60, 0.65)	<0.001	0.99 (0.95, 1.04)	0.812	0.66 (0.63, 0.69)	<0.001
**Cholelithiasis**	0.93 (0.92, 0.95)	<0.001	0.61 (0.60, 0.62)	<0.001	0.95 (0.93, 0.97)	<0.001	0.64 (0.62, 0.65)	<0.001
**Gallbladder and biliary cancer**	0.97 (0.89, 1.06)	0.659	0.83 (0.75, 0.91)	<0.001	0.98 (0.89, 1.07)	0.793	0.86 (0.78, 0.94)	0.003
**Liver diseases**								
**Non-alcoholic fatty liver disease**	0.85 (0.83, 0.88)	<0.001	0.50 (0.49, 0.52)	<0.001	0.87 (0.84, 0.89)	<0.001	0.52 (0.50, 0.54)	<0.001
**Liver cirrhosis**	0.81 (0.77, 0.85)	<0.001	0.75 (0.71, 0.78)	<0.001	0.81 (0.78, 0.85)	<0.001	0.75 (0.71, 0.79)	<0.001
**Liver cancers**	0.79 (0.71, 0.88)	<0.001	0.76 (0.68, 0.85)	<0.001	0.79 (0.71, 0.88)	<0.001	0.75 (0.67, 0.85)	<0.001
**Appendix diseases**								
**Appendicitis**	1.01 (0.96, 1.05)	0.803	0.96 (0.92, 1.01)	0.142	1.01 (0.96, 1.06)	0.812	0.97 (0.92, 1.02)	0.197

[1] fully adjusted model

[2] Two-sided FDR-adjusted P value (Q value) < 0.05 were considered significant.

Table S12. Sensitivity analysis for associations of grip strength and skeletal muscle mass and risk of twenty-four gastrointestinal diseases excluding individuals with missing covariates included in the fully adjusted model (n=24,646)[1, 2]

	Grip strength		Skeletal muscle mass	
	HR (95% CI)	*Q*	HR (95% CI)	*Q*
**Esophagus**				
Barrett's esophagus	**0.90 (0.87, 0.93)**	**<0.001**	**0.87 (0.84, 0.90)**	**<0.001**
Gastroesophageal reflux disease	**0.91 (0.90, 0.92)**	**<0.001**	**0.82 (0.81, 0.83)**	**<0.001**
Esophageal cancer	0.99 (0.92, 1.06)	0.78	**0.91 (0.85, 0.97)**	**0.009**
**Stomach and bowel**				
Gastritis and duodenitis	**0.89 (0.88, 0.90)**	**<0.001**	**0.91 (0.90, 0.93)**	**<0.001**
Celiac disease	**0.87 (0.82, 0.92)**	**<0.001**	**1.25 (1.19, 1.31)**	**<0.001**
Crohn's disease	**0.91 (0.85, 0.98)**	**0.022**	0.95 (0.89, 1.03)	0.209
Intestinal diverticular disease	**0.94 (0.90, 0.99)**	**0.044**	**1.01 (0.96, 1.07)**	**0.589**
Ulcerative colitis	**0.94 (0.93, 0.95)**	**<0.001**	**0.84 (0.83, 0.85)**	**<0.001**
Irritable bowel syndrome	**0.84 (0.82, 0.86)**	**<0.001**	**0.96 (0.94, 0.99)**	**0.003**
Peptic ulcer	**0.84 (0.82, 0.87)**	**<0.001**	**0.86 (0.84, 0.89)**	**<0.001**
Gastric cancer	1.03 (0.96, 1.11)	0.533	**0.94 (0.86, 1.01)**	**0.11**
Small intestinal cancer	0.96 (0.85, 1.10)	0.683	**0.81 (0.71, 0.93)**	**0.005**
Colorectal cancer	1.01 (0.98, 1.04)	0.652	**0.92 (0.90, 0.95)**	**<0.001**
**Pancreas**				
Acute pancreatitis	**0.91 (0.86, 0.96)**	**0.001**	**0.67 (0.63, 0.71)**	**<0.001**
Chronic pancreatitis	**0.86 (0.78, 0.96)**	**0.011**	**0.88 (0.79, 0.99)**	**0.032**
Pancreatic cancer	1.01 (0.95, 1.07)	0.793	**0.90 (0.85, 0.96)**	**0.003**
**Gallbladder and biliary**				
Cholangitis	**0.91 (0.85, 0.98)**	**0.022**	**0.75 (0.70, 0.81)**	**<0.001**
Cholecystitis	0.98 (0.94, 1.02)	0.443	**0.63 (0.60, 0.66)**	**<0.001**
Cholelithiasis	**0.93 (0.92, 0.95)**	**<0.001**	**0.61 (0.60, 0.62)**	**<0.001**
Gallbladder and biliary cancer	0.97 (0.89, 1.06)	0.652	**0.83 (0.76, 0.92)**	**<0.001**
**Liver diseases**				
Non-alcoholic fatty liver disease	**0.85 (0.83, 0.88)**	**<0.001**	**0.51 (0.49, 0.52)**	**<0.001**
Liver cirrhosis	**0.81 (0.77, 0.85)**	**<0.001**	**0.75 (0.71, 0.79)**	**<0.001**
Liver cancers	**0.80 (0.72, 0.89)**	**<0.001**	**0.77 (0.69, 0.86)**	**<0.001**
**Appendix diseases**				
Appendicitis	1.01 (0.96, 1.06)	0.793	0.97 (0.92, 1.01)	0.167

CI, confidence interval; HR, hazard ratio

[1] adjusted for age, sex, ethnicity, Townsend deprivation index, education, healthy diet, alcohol consumption, physical activity, BMI, smoking status and Charlson comorbidity index.
[2] Two-sided FDR-adjusted P value (Q value) < 0.05 were considered significant.

Table S13. Associations of grip strength and skeletal muscle mass and risk of twenty-four gastrointestinal diseases among propensity score-matched population [1, 2]

	Grip strength		Skeletal muscle mass	
	HR (95% CI)	*Q*	HR (95% CI)	*Q*
**Esophagus**				
Barrett's esophagus	**0.92 (0.89, 0.95)**	**<0.001**	**0.86 (0.83, 0.90)**	**<0.001**
Gastroesophageal reflux disease	**0.90 (0.88, 0.91)**	**<0.001**	**0.82 (0.80, 0.83)**	**<0.001**
Esophageal cancer	0.96 (0.90, 1.04)	0.39	**0.88 (0.82, 0.95)**	**0.002**
**Stomach and bowel**				
Gastritis and duodenitis	**0.89 (0.88, 0.90)**	**<0.001**	**0.91 (0.89, 0.92)**	**<0.001**
Celiac disease	**0.85 (0.80, 0.90)**	**<0.001**	**1.28 (1.21, 1.36)**	**<0.001**
Crohn's disease	**0.86 (0.80, 0.93)**	**<0.001**	0.94 (0.87, 1.01)	0.092
Intestinal diverticular disease	**0.94 (0.93, 0.95)**	**<0.001**	**0.83 (0.82, 0.84)**	**<0.001**
Ulcerative colitis	0.96 (0.91, 1.02)	0.25	1.03 (0.97, 1.08)	0.36
Irritable bowel syndrome	**0.80 (0.78, 0.83)**	**<0.001**	**0.92 (0.89, 0.95)**	**<0.001**
Peptic ulcer	**0.84 (0.82, 0.87)**	**<0.001**	**0.83 (0.81, 0.86)**	**<0.001**
Gastric cancer	1.04 (0.96, 1.12)	0.45	**0.91 (0.84, 0.99)**	**0.039**
Small intestinal cancer	0.97 (0.83, 1.13)	0.73	**0.84 (0.72, 0.98)**	**0.037**
Colorectal cancer	1.02 (0.99, 1.05)	0.32	**0.94 (0.91, 0.97)**	**<0.001**
**Pancreas**				
Acute pancreatitis	**0.92 (0.87, 0.98)**	**0.008**	**0.66 (0.62, 0.70)**	**<0.001**
Chronic pancreatitis	**0.82 (0.73, 0.93)**	**0.003**	**0.86 (0.76, 0.98)**	**0.027**
Pancreatic cancer	0.98 (0.92, 1.05)	0.67	**0.91 (0.85, 0.98)**	**0.011**
**Gallbladder and biliary**				
Cholangitis	**0.87 (0.81, 0.93)**	**<0.001**	**0.69 (0.64, 0.74)**	**<0.001**

Cholecystitis	0.97 (0.92, 1.02)	0.31	**0.61 (0.58, 0.64)**	**<0.001**
Cholelithiasis	**0.93 (0.91, 0.95)**	**<0.001**	**0.60 (0.59, 0.62)**	**<0.001**
Gallbladder and biliary cancer	0.94 (0.85, 1.03)	0.25	**0.85 (0.77, 0.94)**	**0.003**
**Liver diseases**				
Non-alcoholic fatty liver disease	**0.81 (0.78, 0.84)**	**<0.001**	**0.52 (0.50, 0.54)**	**<0.001**
Liver cirrhosis	**0.76 (0.71, 0.80)**	**<0.001**	**0.67 (0.63, 0.72)**	**<0.001**
Liver cancers	**0.82 (0.73, 0.93)**	**0.002**	**0.78 (0.69, 0.88)**	**<0.001**
**Appendix diseases**				
Appendicitis	1.01 (0.96, 1.06)	0.82	0.95 (0.91, 1.00)	0.064

CI, confidence interval; HR, hazard ratio

[1] adjusted for age, sex, ethnicity, Townsend deprivation index, education, healthy diet, alcohol consumption, physical activity, BMI, smoking status and Charlson comorbidity index.

[2] Two-sided FDR-adjusted P value (Q value) < 0.05 were considered significant.

Table S14. ICD codes for identification of disease outcomes of interest

Gastrointestinal diseases	ICD-9	ICD-10	Reference
**Esophagus**			
Barrett's esophagus	5302	K22.7	PMID: 17229223, 28600361, 30548214
Gastroesophageal reflux disease	53011, 53081	K21	PMID: 17229223, 30548214
Esophageal cancer	150	C15	PMID: 31792601
**Stomach and bowel**			
Gastritis and duodenitis	535	K29	PMID: 27194488
Celiac disease	5790	K90.0	PMID: 27501017
Crohn's disease	555	K50	PMID: 27501017
Ulcerative colitis	556	K51	PMID: 27501017
Intestinal diverticular disease	562	K57	PMID: 34139333
Irritable bowel syndrome	5641	K58	PMID: 27501017, 31567167
Peptic ulcer	531-534	K25-K28	PMID: 32628718, 28503076, 32994689
Gastric cancer	151	C16	PMID: 20551458
Small intestinal cancer	152	C17	PMID: 31040384
Colorectal cancer	153, 1540, 1541	C18-C20	PMID: 22510213, 33675346
**Pancreas**			
Acute pancreatitis	5770	K85	PMID: 34129395
Chronic pancreatitis	5771	K86.0, K86.1	PMID: 30039239
Pancreatic cancer	157	C25	PMID: 21346976
**Gallbladder and biliary**			
Cholangitis	5761	K83.0	PMID: 27194488
Cholecystitis	575.0, 575.1	K81	PMID: 27194488
Cholelithiasis	574.0, 574.1, 574.2	K80	PMID: 27194488
Gallbladder and biliary cancer	156	C23, C24	PMID: 27516528, 31339558

**Liver**			
Non-alcoholic fatty liver disease	5718, 5719	K76.0, K75.8	PMID: 26274335
Cirrhosis	5715, 5712, 4560, 4561, 4562, 5723, 5724, 5722,	K74.6, K70.3, I85, I98.2, I98.3, K76.6, K72.9, K76.7	PMID: 35166399
Liver cancers	155	C22	PMID: 23182222, 23489585, 31339558
**Appendix**			
Appendicitis	540-542	K35-K37	

ICD, international classification of disease

Table S15. Diagnostic codes (Read codes) for identification of outcomes of interest in primary care data

See supplementary file uploaded as Excel file

Table S16. Definitions for covariates

	UK Biobank	Missing rate
Age at recruitment	This is a derived variable based on the date of birth and date of attending an initial assessment center and refers to the age of the participant on the day they attended an Initial Assessment Centre, truncated to the whole year.<br>(variable handling: as a continuous variable)	0
Sex	A mixture of the sex the National Health Service had recorded for the participant and self-reported sex.<br>(variable handling: categorical variable "**Female**", "**Male**")	0
Ethnicity	Self-reported: "What is your ethnic background". We classified the variable into: **White** (White) and **Others** (Mixed, Asian or Asian British, Black or Black British, Chinese, and other ethnic groups) because the number of non-white ethnic backgrounds was too small	0.32%
Education	Self-reported: "Which of the following qualifications do you have" We classified the variable into **College** (College or University degree) and **Below college** (A levels/AS levels or equivalent, O levels/GCSEs or equivalent, CSEs or equivalent, NVQ or HND or HNC or equivalent, other professional qualifications eg: nursing, teaching, and none of the above) based on previous literature studying inflammatory bowel disease in the UK Biobank	0.11%
Townsend deprivation index (TDI)	The higher, the more socioeconomic deprivation one was suffering. TDI was derived according to the unemployment rate, the percentage of overcrowded households, the percentage of people without cars, and the percentage of people without houses for each area in the UK, and baseline TDI was calculated immediately before the participant joined UK Biobank based on the preceding national census output areas. Each participant was assigned a score corresponding to the output area in which their postcode is located<br>(variable handling: as continues variable)	0.75%
Smoking status	Self-reported current/past smoking status of the participant. We classified the variable handlings into **Never smoked** (Never) and **Former or current smoker** (Previous, current)	0.29%
Alcohol consumption	Participants self-reported the number of alcohol units (10 ml of pure ethanol) consumed, in "units per week" (for frequent drinkers) or "units per month" (for less frequent drinkers), across several beverage categories (red wine, white wine/champagne, beer/cider, spirits, fortified wine, and "other"). To calculate alcohol consumption as per	0.53%

	UK Biobank	Missing rate
	guidelines, multiply the volume by the alcohol content in percent and divide by drink-equivalent; then convert to grams:<br>1 drink equivalent described as containing 14g of pure alcohol.<br>125ml wine=0.85 drink-equivalents,<br>4% ABV pint beer = 1.28 drink-equivalents,<br>25ml spirits=0.57 drink-equivalents,<br>50ml fortified wine= 0.56 drink-equivalents,<br>None to moderate level of alcohol consumption was defined as 0-14 g/d for women and 0-28 g/d for men according to US dietary guidelines, which is defined as a heavy level<br>(variable handling: categorical variable "**None to moderate**", "**Heavy**")	
Physical activity	Self-reported: UK Biobank physical activity questionnaire (IPAQ short form). We classified the variable handlings into **Adequate** (150 minutes moderate activity per week OR ≥ 75 minutes vigorous activity per week OR equivalent combination OR moderate physical activity at least 5 days a week or vigorous activity once a week) and **Inadequate** (below adequate level) recommended by the American Heart Association.	3.2%
Body mass index (BMI)	BMI value is constructed from height and weight measured during the initial assessment center visit. The relevant variable was measured by trained staff.<br>(variable handling: as continues variable)	0.52%
Healthy diet	Self-reported UK Biobank food frequency questionnaire (FFQ). Lourida et al developed the variable reflecting a healthy diet according to this recommendation in the UK Biobank using FFQ. FFQ collected the following items either quantitatively or as the frequency of intake (e.g., 2-4 times/week). For the variables recorded in the frequency, we assigned them with mean values to get a quantitative estimate (e.g., 2-4 times/week-> 3 servings/week).<br>At least 4 of the following 7 food groups:<br>1. Fruits: ≥ 3 servings/day (items in FFQ: dried fruit, fresh fruit)<br>2. Vegetables: ≥ 3 servings/day (items in FFQ: cooked vegetable, raw or salad vegetable)<br>3. Fish: ≥2 servings/week (items in FFQ: oily fish, non-oily fish)<br>4. Processed meats: ≤ 1 serving/week (items in FFQ: processed meat)<br>5. Unprocessed red meats: ≤ 1.5 servings/week (items in FFQ: unprocessed pork, beef, mutton)<br>6. Whole grains: ≥ 3servings/day (items in FFQ: cereal intake, wholemeal or wholegrain bread)	1.0%

	**UK Biobank**	**Missing rate**
	7. Refined grains: ≤1.5servings/day (items in FFQ: other bread intakes)<br>(variable handling: as a categorical variable, "**Healthy**", "**Unhealthy**")	
Charlson Comorbidity Index (CCI)	CCI is a highly cited and well-established tool for measuring comorbidity in clinical research. We calculated CCI as a variable reflecting objective heath status (range: 0–16). Mak JKL et al developed the calculation of CCI in the UK Biobank, which were constructed based on 17 comorbidities (myocardial infarction, congestive heart failure, peripheral vascular disease, cerebral vascular disease, dementia, pulmonary disease, connective tissue disorder, peptic ulcer, liver disease, diabetes, diabetes complications, paraplegia, renal disease, cancer, metastatic cancer, severe liver disease, and acquired immune deficiency syndrome) with assigned weights associated with ICD codes from hospital records. (variable handling: as continues variable range from 0 to 16)	0
INFLA-score	To compute the INFLA-score, all four components, laying in the highest deciles (7th to 10th) were assigned values from +1 to +4; while biomarker levels laying in the lowest deciles (1st to 4th) were given values from -4 to -1.	7.34%
